# In pursuit of synergy: An investigation of the PI3K/mTOR/MEK co-targeted inhibition strategy in NSCLC

**DOI:** 10.18632/oncotarget.12755

**Published:** 2016-10-19

**Authors:** Susan Heavey, Sinead Cuffe, Stephen Finn, Vincent Young, Ronan Ryan, Siobhan Nicholson, Niamh Leonard, Niall McVeigh, Martin Barr, Kenneth O'Byrne, Kathy Gately

**Affiliations:** ^1^ Department of Clinical Medicine, Trinity College Dublin, Dublin, Ireland; ^2^ Department of Cardiothoracic Surgery, St. James's Hospital, Dublin, Ireland; ^3^ Department of Histopathology, St James Hospital, Dublin, Ireland; ^4^ Cancer and Ageing Research Program, Institute of Health and Biomedical Innovation at the Translational Research Institute (TRI), Queensland University of Technology, Brisbane, Australia

**Keywords:** PI3K, MEK, NSCLC, lung, co-target

## Abstract

Clinical PI3K inhibition has been somewhat disappointing, due to both inadequate patient stratification and compensatory cell signalling through bypass mechanisms. As such, investigation of PI3K-MEK co-targeted inhibition has been recommended. With high mortality rates and a clear need for new therapeutic intervention strategies, non-small cell lung cancer (NSCLC) is an important setting to investigate the effectiveness of this approach.

Here, 174 NSCLC tumours were screened for 150 mutations by Fluidigm technology, with 15 patients being profiled for phosphoprotein expression. The effects of GDC-0941 (a pan PI3K inhibitor), GDC-0980 (a dual PI3K/mTOR inhibitor) and GDC-0973 (a MEK inhibitor) alone and in combination were assessed in 3 NSCLC cell lines.

PIK3CA was mutated in 5.17% of NSCLC patients. GDC-0941 and GDC-0980 treatment induced anti-proliferative and pro-apoptotic responses across all NSCLC cell lines, while GDC-0973 treatment induced only anti-proliferative responses. GDC-0980 and GDC-0973 combined treatment led to significant increases in apoptosis and synergistic reductions in proliferation across the panel of cell lines.

This study found that the PI3K/MEK co-targeted inhibition strategy is synergistic in all 3 molecular subtypes of NSCLC investigated. Consequently, we would advocate clinical trials for NSCLC patients combining GDC-0980 and GDC-0973, each of which are separately under clinical investigation currently.

## INTRODUCTION

### Lung cancer incidence and treatment strategies

In 2013, lung cancer was the leading cause of cancer related death in men, accounting for 1.3 million male deaths worldwide, along with 535,000 female deaths [1]. This study focuses on Non-Small Cell Lung Cancer (NSCLC), which accounts for 80% of lung cancers.

Several key “driver” genes are mutated in NSCLC including, epidermal growth factor receptor (EGFR), anaplastic lymphoma kinase (ALK), KRAS, human epidermal growth factor receptor 2 (HER2), v-raf murine sarcoma viral oncogene homolog B1 (BRAF), MET, phosphatidylinositol 3-kinase catalytic α (*PIK3CA*), AKT and mitogen-activated protein kinase kinase 1 (*MAP2K1*). These aberrant genes are obvious targets for inhibition.

### PI3K signalling and aberrations

PI3K-AKT-mTOR signalling is known to play a role in carcinogenesis and tumour progression, and mutations have been reported in *PIK3CA* in a range of human cancers. These *PIK3CA* mutations most frequently affect residues Glu542 and Glu545 encoding the catalytic domain, and His1047 encoding the kinase domain [2]. *PIK3CA* mutation rates vary widely across different cancer types, being reported in 2-7% of lung cancers but up to 40% of breast and colorectal cancers [3, 4]. It should be noted that even though mutation rates are lower in lung cancer, this would still represent 32,000 – 112,000 patients diagnosed annually, and as such any therapeutic intervention strategies based on this mutation would be applicable to a large cohort of patients. Activation of mTOR, AKT and loss of PTEN have each been associated with a poorer prognosis in lung cancer patients [5, 6], however an association between *PIK3CA* mutation status and prognosis has been controversial [6]. It has been hypothesized that a portion of *PIK3CA*-mutant lung cancers could be dependent on *PIK3CA* as a driver oncogene, whereas in other cases, the *PIK3CA* mutation may modulate the effect of another oncogenic process [7].

PI3K pathway signalling is complex, with multiple growth factor receptors capable of activating the pathway. Activation of this pathway is associated with resistance to chemotherapy, immunotherapy and targeted therapies [4], and as such it is crucial that improved targeting strategies are developed to thwart PI3K signalling. One method of achieving this may be the utilization of horizontal or vertical co-targeted inhibition strategies. AKT-mTOR signalling can be activated due to the extensive cross-talk with other intracellular signalling pathways and multiple levels of redundancy, as well as mutation in *AKT* or loss of PTEN. As such it is plausible that the pathway can be activated in patients in the absence of *PIK3CA* mutation, as has been seen in breast cancer [8]. Moving forward, patient stratification through alternate methods may be preferable to the *PIK3CA* mutation approach, such as expression of key PI3K pathway proteins mTOR, pAKT or loss of PTEN.

### Targeting the PI3K pathway in lung cancer

Activation of the PI3K-AKT-mTOR pathway is associated with all eight hallmarks of cancer, and has been shown to correlate with a poorer prognosis in NSCLC and other cancers [4, 5]. Inhibitors of class I PI3Ks, AKT and mTOR have been investigated in both the laboratory and clinical settings, and more recently dual PI3K-mTOR inhibitors and isoform specific PI3K inhibitors have been developed and are in early stage clinical trials [4]. Since the PI3K-AKT-mTOR can also become activated in the absence of *PIK3CA* mutation, for example through loss of PTEN, or mutations in *AKT* and *MTOR*, it is hoped that the clinical activity of PI3K inhibitors may not be limited to *PIK3CA* mutant tumours. In September 2015, the results of a Phase II trial of pan-PI3K inhibitor Buperlisib were published, where the trial did not meet its primary objective. Here it was determined that PI3K activation may not be the oncogenic driver in NSCLC, and as such PI3K inhibition may not be effective as a monotherapy in this setting. The authors recommend a co-targeted inhibition approach moving forward, where other oncogenic pathways are targeted alongside the PI3K pathway in NSCLC [9].

A number of pan-Class I PI3K inhibitors have been investigated in both the laboratory and clinical settings, with varying levels of success. GDC-0941 is a pan PI3K inhibitor which will be investigated in this study, and which is currently being investigated in clinical trials [10, 11]. Preclinical data has supported *PIK3CA* mutations and PTEN loss as predictive biomarkers of response to the drug in lung, breast, and other solid tumours [12–14]. To date, 13 trials involving GDC-0941 are ongoing, mainly in NSCLC and breast cancer.

Dual targeted inhibition of PI3K and mTOR is hoped to provide superior inhibition of PI3K pathway signalling, as this strategy can prevent feedback activation of mTOR in response to PI3K inhibition. GDC-0980 is a dual PI3K-mTOR inhibitor which is investigated in-depth in this study. The drug demonstrated excellent downstream inhibition of the PI3K pathway *in vitro*, with the strongest effects being observed in lung, breast and prostate cancer cell lines [15]. There are 12 trials ongoing for this drug, with Phase I trials in solid tumours and Phase II studies in endometrial carcinoma, renal cell carcinoma, prostate cancer and breast cancer.

One major mechanism of resistance to targeted inhibition lies in the ability of a cancer cell to utilise an alternative pathway as a bypass track, to maintain or regain oncogenic signalling. Several strategies have been investigated in order to pre-empt this mechanism of resistance, most of which involve co-targeting the PI3K pathway with either a growth factor receptor, or an alternative intracellular signalling protein. *KRAS* is commonly mutated in NSCLC (~20%-30% in adenocarcinoma,[16]), with an estimated 70% of lung tumours displaying RAS-RAF-MAPK pathway activation [17]. Since *KRAS* activation stimulates signalling through both the PI3K and MEK pathways, there have been several studies investigating the efficacy of co-targeting the PI3K and MEK pathways in NSCLC [18]. A phase I trial is currently recruiting patients with solid tumours for combination treatment with PI3K pathway inhibitor BKM120 and MAPK pathway inhibitor MEK162 [19]. A Phase I trial has recently been completed [20] which investigated the combination of GDC-0941 with GDC0973 in solid tumours. There has been no study to date in the clinical setting combining GDC-0980 with GDC-0973, which we hypothesize may provide a superior blockade of oncogenic signalling. One previous publication has investigated the combination of these two drugs *in vivo*, where it was observed that intermittent dosing of the two drugs led to increased anti-tumour efficacy in a melanoma xenograft model [21].

## RESULTS

### PI3K pathway activation in NSCLC patients

DNA was isolated from 175 NSCLC FFPE patient samples and screened for 150 clinically relevant mutations in 13 genes using Fluidigm technology. The genes included were: EGFR, PIK3CA, KRAS, BRAF, NRAS, AKT1, FLT3, HRAS, KIT, MET, JAK2, MYD88 and ERBB2. PIK3CA was mutated in 5.17% of patient samples (9 patients; 4: E545K, 2: H1047R, 1: H1047L, 1: G1049R and 1: E542K). None of these 9 patients bore concomitant mutations except for 1 patient who bore a PIK3CA mutation (H1047R) and an EGFR mutation (L858R).

**Figure 1:**
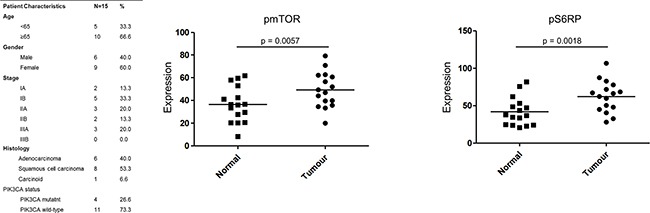
PI3K activation in NSCLC patients Phosphorylation of mTOR and pS6RP was profiled in 15 matched tumour and normal NSCLC patient fresh tissue samples Intensity of expression was quantified using Image J. A Wilcoxon signed rank test was performed to compared tumour versus normal mean expression values.

PIK3CA mutation status was not found to correlate with survival (median survival 1147 days in wild-type versus 1149 days in mutant, p=0.9697). Protein was isolated from 15 NSCLC patient matched tumour and normal fresh tissue samples and profiled for phosphoprotein expression using PathScan intracellular signalling arrays. Both pmTOR and pS6RP were significantly more highly expressed in tumour tissue than matched normal tissue samples (Figure 1). 4 of these 15 patients harboured a mutation in PIK3CA, which did not correlate with phosphoprotein expression.

### Histological and molecular subtype of NSCLC cell lines used in this study

**Figure 2:**
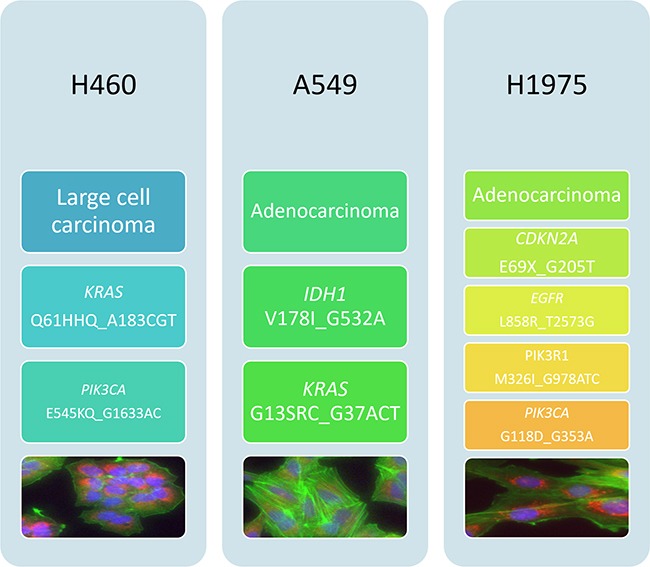
Histological and mutational characterisation of NSCLC cell lines NSCLC cell lines used in this study were chosen based on their differing histological subtypes. Mutation status was obtained from the COSMIC database. Cell images were obtained using the InCell 1000. Each cell line was stained with Höechst blue nuclear stain, mitotracker red mitochondrial stain and phalloidin green F-actin stain.

Cell lines used in this study were chosen to represent different histological and molecular subtypes of NSCLC. H460 is a large cell carcinoma cell line while A549 and H1975 are adenocarcinoma cell lines. Molecular characteristics of available NSCLC cell lines as published in the COSMIC (Catalogue Of Somatic Mutations In Cancer) database were taken into account when selecting the panel. Based on COSMIC data, the H460 cell line bares mutations in both *PIK3CA* and *KRAS*, the A549 cell line bares wild-type *PIK3CA* and mutated *KRAS* and the H1975 cell line bares mutated PIK3CA and wild-type KRAS [22–24]. Cell morphology was observed by fluorescence microscopy. Cell lines were stained using Höechst nuclear stain (blue) Mitotracker mitochondrial stain (red) and phalloidin F-actin stain (green) and imaged using the InCell 1000 (Figure 2).

### Gene expression in NSCLC cell lines

Gene expression was compared across H460, A549 and H1975 cells using two gene expression arrays (SABiosciences): one which profiles the expression of genes related to mTOR signalling, and one which profiles the expression of genes related to cancer drug resistance.

H460 and A549 cell lines had similar levels of *PIK3CA*, but H460 cells expressed higher levels of *AKT1S1* (165.1 fold), *AKT2* (5.42 fold), *PTEN* (3634.11 fold), *GSK3B* (3.93 fold), *MTOR* (951.67 fold) and *MAPK1* (1717.71 fold) than A549 cells, and lower levels of *AKT3* (−36.56 fold) among others (Supplementary Figure S1). Similarly, H1975 cells expressed higher levels of *PIK3CA* (2.33 fold), *AKT1S1* (130.99 fold), *AKT2* (3.48 fold), *PTEN* (1055.37 fold), *GSK3B* (6.12 fold), *MTOR* (1015.72 fold) and *MAPK1* (2447.82 fold) than A549 cells, and lower levels of *AKT3* (−4396.99 fold) than A549 cells (Supplementary Figure S2). H1975 cells expressed higher levels of *PIK3CA* (2.44 fold) and *DEPTOR* (6.01 fold) than H460 cells, but expressed lower levels of *AKT3* (−120.27 fold), *PTEN* (−3.44 fold) and similar levels of *AKT1S1* (−1.26 fold), *AKT2* (−1.56 fold), *GSK3B* (1.56 fold), *MTOR* (1.07 fold) and *MAPK1* (1.43 fold) as H460 cells (Supplementary Figure S3).

H460 cells expressed higher levels of *ABCB1* (250.43 fold) and several other cancer drug resistance genes than A549 cells, but in general most of the human cancer drug resistance genes included were expressed more highly in A549 cells than H460 cells (Supplementary Figure S4). H1975 cells expressed higher levels of *CDKN2A* (362.66 fold) and several other cancer drug resistance genes than A549 cells, but again most genes on the panel were more highly expressed in A549 cells than H1975 cells (Supplementary Figure S5). H1975 cells also expressed higher levels of *CDKN2A* (1141.35 fold) than H460 cells, and expressed higher levels of *ESR2* (119.11 fold) and lower levels of *ESR1* (−2221.47 fold) than H460 cells (Supplementary Figure S6).

**Figure 3:**
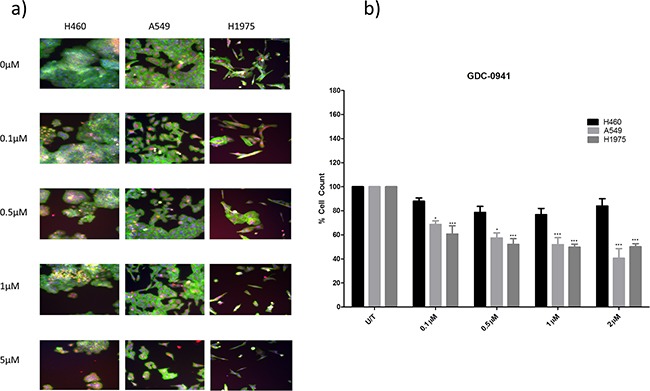
The effect of GDC-0941 treatment on viability in a panel of NSCLC cells H460, A549, and H1975 cells were treated at noted concentrations of GDC-0941 for 48 hours in a 96 well plate and then fixed & stained with Höechst nuclear stain, mitotracker red mitochondrial stain and phalloidin green F-actin stain. **A.** Stained plates were imaged using the InCell 1000 (10X objective, 10 fields per well, triplicate wells, n=3). **B.** Images were analysed for cell count using InCell 1000 software. Cell numbers from each treatment group were compared with untreated cell numbers and are represented here as % cell count compared to untreated cells, with mean ± SEM for treated groups. */***: p<0.05/0.001 respectively, compared to untreated cells of the same cell line.

A number of genes were reported as having very large fold difference between cell lines, for example PPP2CA (7606.65 fold), EIF4EBP1 (2706.94 fold) and RHOA (3078.48 fold) (Figure 3). In such cases these genes are noted with an ‘A’ which refers to one out of the two samples not expressing the gene (or expressing it after the cycle threshold of 30 cycles). As such the gene is expressed in one cell line but not the other. The number of genes up/down regulated in each comparison is noted in each figure legend.

### Anti-cancer effects of GDC-0941 and GDC-0980 in *PIK3CA* mutant and wild-type NSCLC cell lines

Two *PIK3CA* mutant cell lines (H460 and H1975) and one *PIK3CA* wild-type cell line (A549) were used in this study. Here, the three cell lines were treated with either GDC-0941 or GDC-0980. Cell viability was measured qualitatively and quantitatively after 48 hours by a multiparameter apoptosis assay (high content analysis), or cell proliferation was measured after 72 hours quantitatively by BrdU proliferation assay.

**Figure 4:**
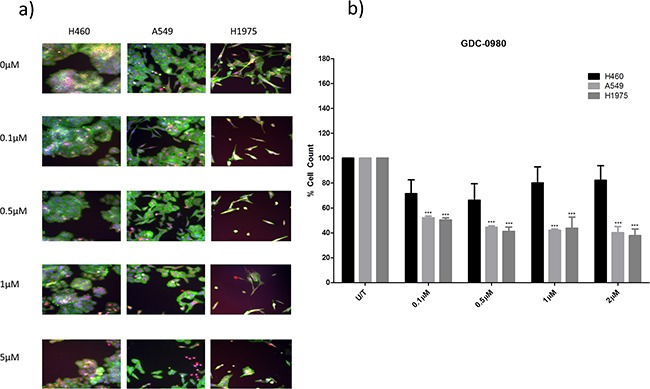
The effect of GDC-0980 treatment on viability in a panel of NSCLC cells H460, A549, and H1975 cells were treated at noted concentrations of GDC-0980 for 48 hours in a 96 well plate and then fixed & stained with Höechst nuclear stain, mitotracker red mitochondrial stain and phalloidin green F-actin stain. **A.** Stained plates were imaged using the InCell 1000 (10X objective, 10 fields per well, triplicate wells, n=3). **B.** Images were analysed for cell count using InCell 1000 software. Cell numbers from each treatment group were compared with untreated cell numbers and are represented here as % cell count compared to untreated cells, with mean ± SEM for treated groups. ***: p<0.001 compared to untreated cells of the same cell line.

**Figure 5:**
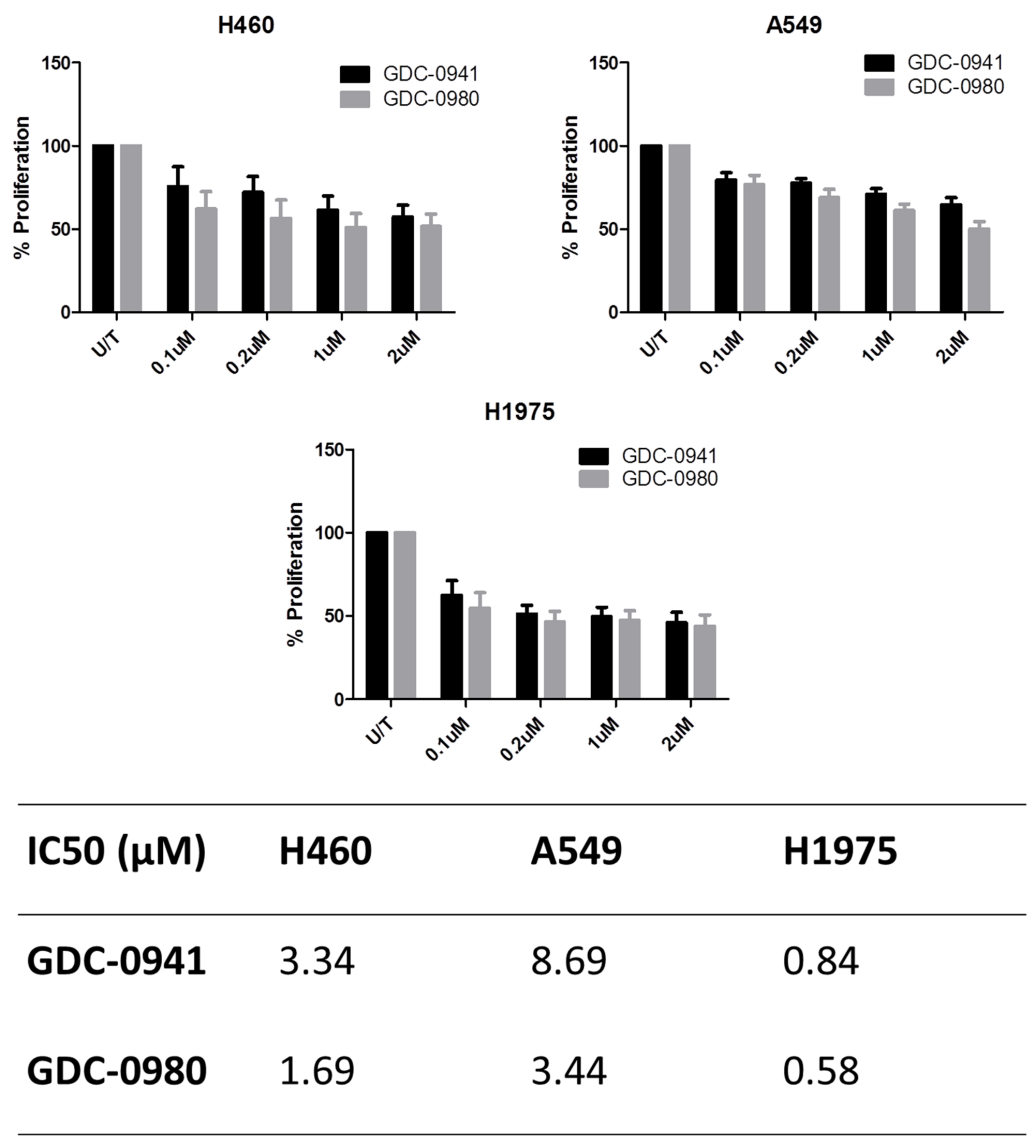
Comparison of the anti-proliferative effects of GDC-0941 and GDC-0980 in NSCLC cell lines Cells were treated at noted concentrations of GDC-0941 or GDC-0980 for 72 hours, then proliferation rates were examined by BrdU incorporation ELISA (triplicate wells, n=3). Raw absorbance values for each treatment group were compared with untreated sample values, which were set to 100%. Data was then expressed as % proliferation compared to untreated cells, with mean ± SEM. Data from a) was analysed by linear regression and IC50 values were determined using GraphPad Prism.

Both GDC-0941 and GDC-0980 were found to induce a reduction in cell viability in all three cell lines, as observed qualitatively (Figure 3a, Figure 4a) and by cell count (Figure 3b, Figure 4b). Both GDC-0941 and GDC-0980 were found to have dose dependant anti-proliferative effects on all three cell lines. H1975 cells were most sensitive to both drugs, and A549 cells were least sensitive to both drugs, as measured by IC50 concentration. There was no statistically significant difference (i.e. p>0.05) between the anti-proliferative effects of the two drugs in any cell line, although the IC50 concentrations were lower for GDC-0980 (Figure 5).

### Anti-cancer effects of GDC-0973 in NSCLC cell lines

**Figure 6:**
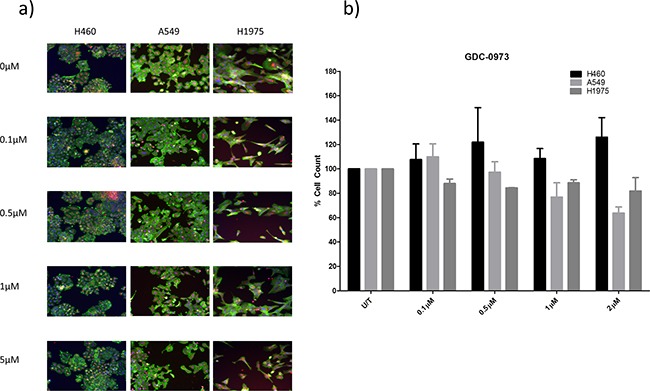
The effect of GDC-0973 treatment on viability in a panel of NSCLC cells H460, A549, and H1975 cells were treated at noted concentrations of GDC-0973 for 48 hours in a 96 well plate and then fixed & stained with Höechst nuclear stain, mitotracker red mitochondrial stain and phalloidin green F-actin stain. **A.** Stained plates were imaged using the InCell 1000 (10X objective, 10 fields per well, triplicate wells, n=3). **B.** Images were analysed for cell count using InCell 1000 software. Cell numbers from each treatment group were compared with untreated cell numbers and are represented here as % cell count compared to untreated cells, with mean ± SEM for treated groups.

**Figure 7:**
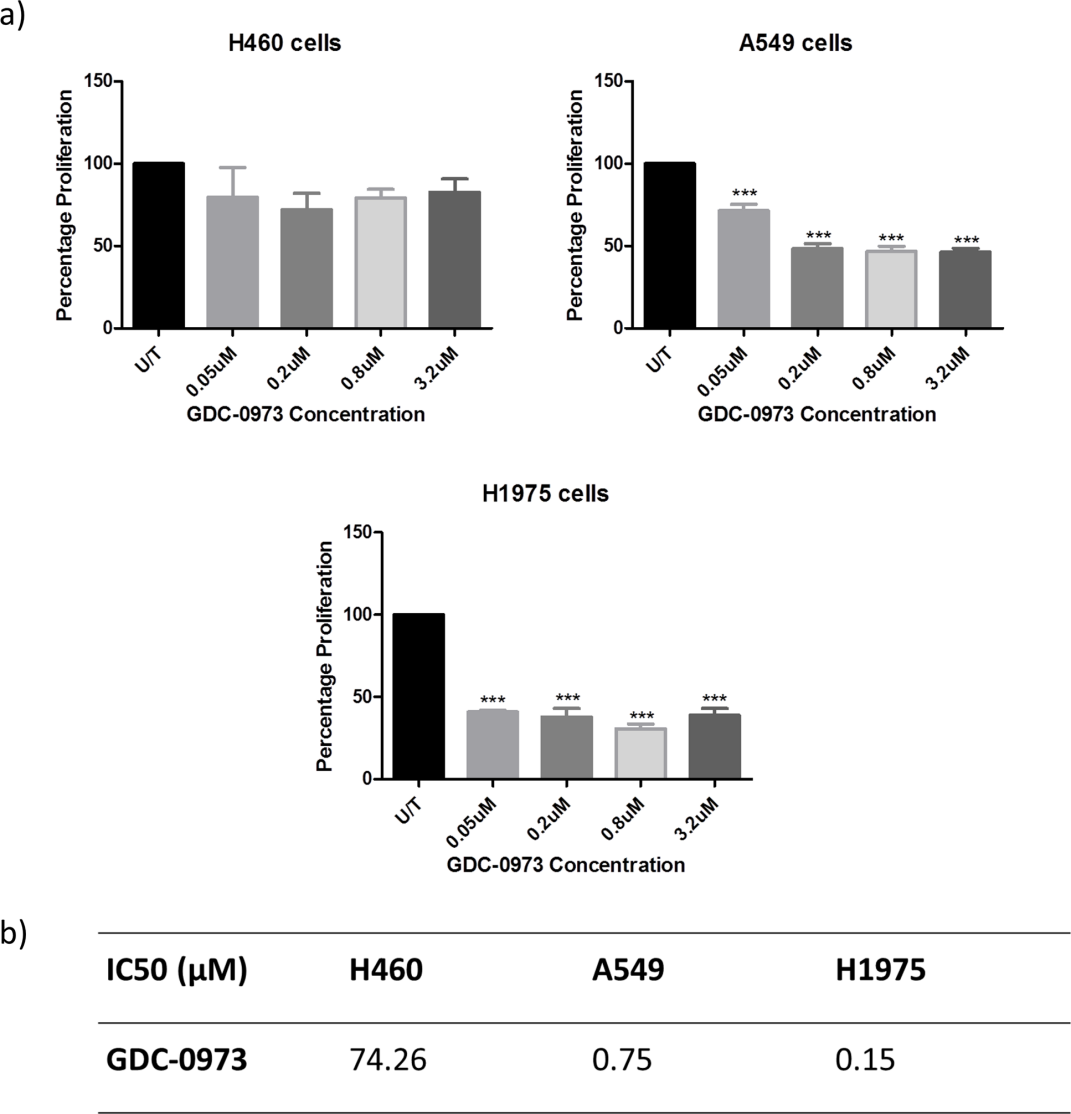
Anti-proliferative effects of GDC-0973 in a panel of NSCLC cell lines Cells were treated at noted concentrations of GDC-0973 for 72 hours, then proliferation rates were examined by BrdU incorporation ELISA (triplicate wells, n=3). **A.** Raw absorbance values for each treatment group were compared with untreated sample values, which were set to 100%. Data was then expressed as % proliferation compared to untreated cells, with mean ± SEM. **B.** Data from a) was analysed by linear regression and IC50 values were determined using GraphPad Prism.

This study will investigate the effects of combining PI3K pathway inhibition with MEK pathway inhibition. As such, the effects of MEK inhibitor GDC-0973 were first investigated alone in H460, A549 and H1975 cells. GDC-0973 did not appear to have a strong effect on viability qualitatively (Figure 6a), and did not induce a statistically significant reduction in cell count in any cell line (Figure 6b). However, GDC-0973 did induce a reduction in proliferation in A549 and H1975 cells (Figure 7).

### Co-targeted inhibition of the PI3K and MEK pathways in NSCLC

Due to the extensive cross-talk and feedback stimulation that is known to exist between the PI3K and MEK pathways, there has been increased interest in co-targeting PI3K and MEK as an anti-cancer treatment. Here, the effects of GDC-0980 and GDC-0973 in combination on viability, proliferation and protein expression were investigated in H460, A549 and H1975 cells.

**Figure 8:**
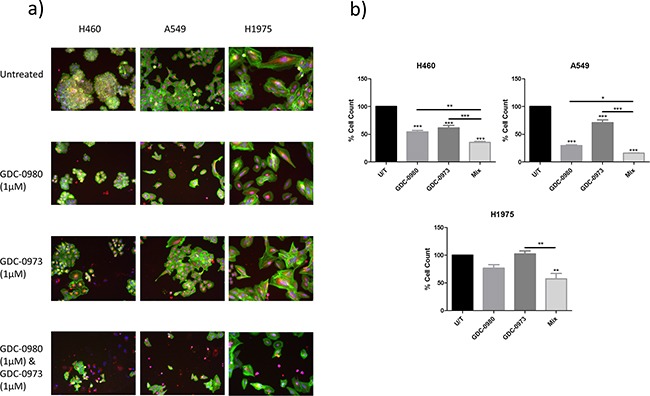
Effects of PI3K-mTOR and MEK co-targeted inhibition on NSCLC cell viability H460, A549 and H1975 cells were treated with GDC-0980 (1μM), GDC-0973 (1μM), both GDC-0980 (1μM) and GDC-0973 (1μM) or left untreated for 48 hours in a 96 well plate and then fixed & stained with Höechst nuclear stain, mitotracker red mitochondrial stain and phalloidin green F-actin stain. **A.** Stained plates were imaged using the InCell 1000 (10X objective, 10 fields per well, triplicate wells, n=3). **B.** Images were analysed for cell count using InCell 1000 software. Treatment groups were compared with untreated cell numbers (raw data) and are represented here as % cell count compared to untreated cells, with mean ± SEM for treated groups. */**/***: p<0.05/0.01/0.001.

The reduction in cell viability induced by GDC-0980 and GDC-0973 in combination was significantly greater than that of either drug alone in H460 and A549 cell lines. A similar trend was observed in H1975 cells, though there was not a statistically significant difference between the effects of GDC-0980 and the mix of the two drugs (Figure 8).

**Figure 9:**
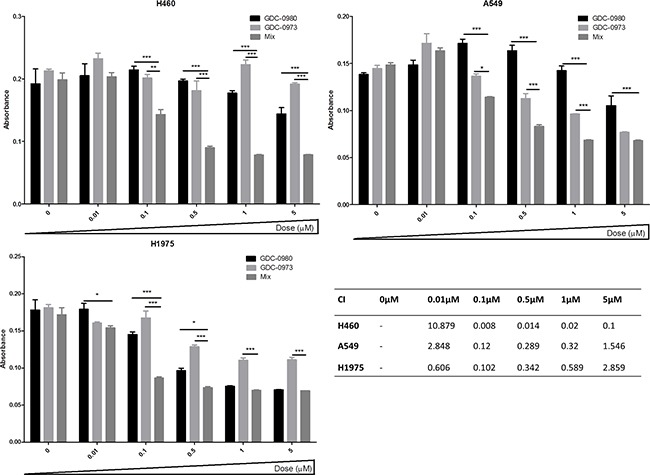
PI3K-mTOR and MEK combined inhibition induces a synergistic reduction in proliferation in NSCLC cells **A.** H460, **B.** A549 and **C.** H1975 cells were treated with GDC-0980, GDC-0973 or a 1:1 mix of GDC-0980 & GDC-0973 at noted concentrations for 72 hours. Proliferation rates were then assessed by BrdU incorportation ELISA (triplicate wells, n=3). Raw absorbance values for each treatment group were compared with untreated sample values, which were set to 100%. Data was then represented as absorbance (which correlates directly with proliferation) with mean ± SEM. **D.** Data was analysed using CalcuSyn software which tests for synergistic, additive or antagonistic drug interactions via the Chou-Talalay method. A combination index (CI) of >1 indicates an antagonistic interaction, CI = 1 indicates an additive interaction and CI < 1 indicates a synergistic interaction. */**/***: *p*<0.05/0.01/0.001 respectively.

A BrdU proliferation assay was used to interrogate the interaction between GDC-0980 and GDC-0973 in the three NSCLC cell lines in more detail. CalcuSyn software was used to analyse the data, returning combination index (CI) values for the drug interaction at each concentration used, based on the Chou-Talalay equation. A CI of <1 implies that there is a synergistic interaction between the two drugs, CI=1 implies an additive effect between the two drugs and a CI>1 implies that there is an antagonistic effect between the two drugs. In H460 cells, the drugs had a synergistic interaction at 0.1μM, 0.5μM, 1μM and 5μM (Figure 9a). In A549 and H1975 cells, there was a synergistic interaction between the two drugs at 0.1μM, 0.5μM and 1μM (CI<1, Figure 9b, Figure 9c).

**Figure 10:**
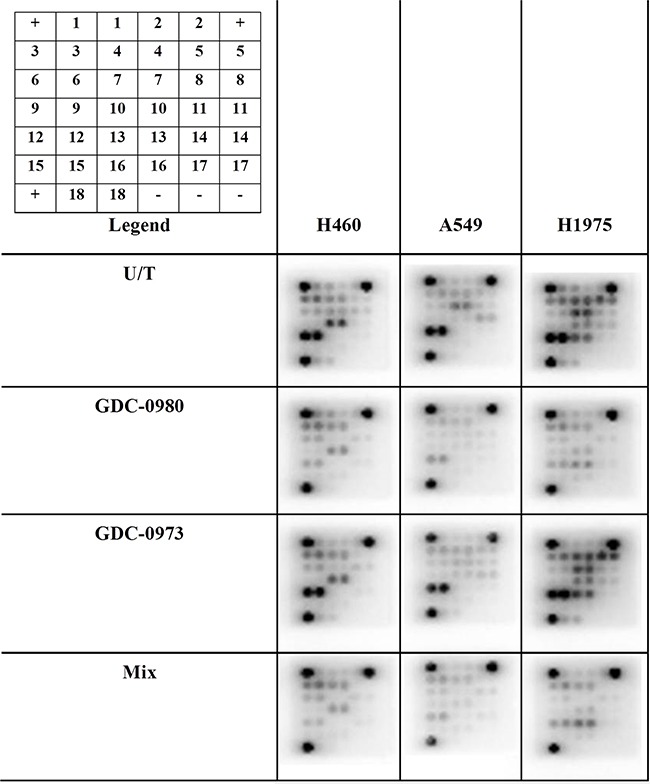
The effects of co-targeting PI3K-mTOR and MEK on phosphoprotein expression in a panel of three NSCLC cell lines Protein was isolated from H460, A549 and H1975 cell lines at baseline and after treatment with GDC-0980 (1μM, 4hrs), GDC-0973 (2μM, 6hrs) and a mix of the 2 drugs using Cell Signalling lysis buffer. Intracellular signalling protein expression was explored using PathScan arrays (duplicate spots, n=3). Array images are shown with postive (+) and negative (−) controls identified. 1: ERK1/2 (Thr202/Tyr204), 2:Stat1(Tyr701), 3: Stat3 (Tyr705), 4: AKT (Thr308), 5: AKT (Ser473), 6: AMPKα (Thr172), 7: S6RP (Ser235/236), 8: mTOR (Ser2448), 9: HSP27 (Ser78), 10: BAD (Ser15), 11: p70S6K (Thr389), 12: PRAS40 (Thr246), 13: p53 (Ser15), 14: p38 (Thr180/Tyr182), 15: SAPK/JNK (Thr183/Tyr185), 16: PARP (Asp214), 17: Caspase-3 (Asp175), 18: GSK-3β (Ser9).

**Figure 11:**
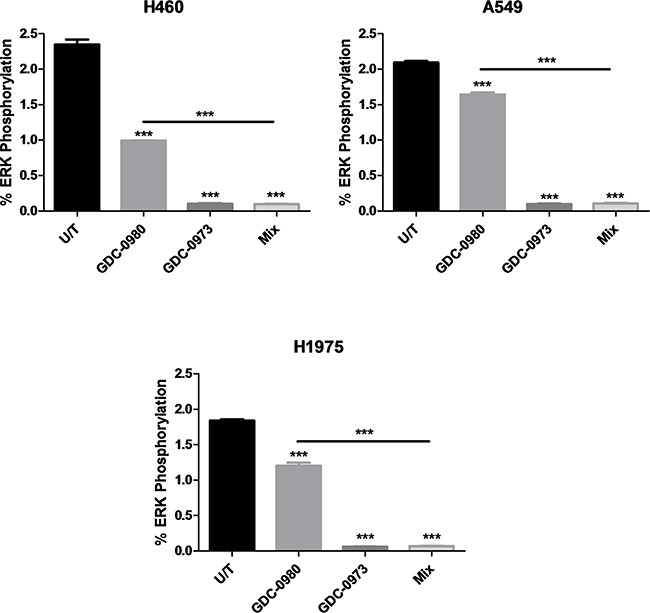
Effect of GDC-0980 and/or GDC-0973 treatment on total ERK and pERK1/2 (Thr202/Tyr204, Thr185/Tyr187) expression in a panel of NSCLC cell lines Protein was isolated from H460, A549 and H1975 cell lines at baseline and after treatment with GDC-0980 (1μM, 4hrs), GDC-0973 (2μM, 6hrs) and a mix of the 2 drugs using MSD lysis buffer. Total ERK and pERK1/2 (Thr202/Tyr204, Thr185/Tyr187) expression was explored using MSD arrays (duplicate wells, n=3). Spot intensity was quantified through MSD software and data were expressed as % Phosphoprotein = ((2 x Phospho-signal) / (Phospho-signal + Total signal)) x 100. Percentage phosphorylation was then expressed as mean ± SEM. ***: p<0.001, compared to untreated cells unless otherwise noted.

Optimal treatment times and concentrations for assessment of protein expression in response to inhibition by GDC-0941, GDC-0980 and GDC-0973 were determined by Western blot (data not shown). PathScan arrays were then used to profile the expression of 18 phosphorylated or cleaved proteins in response to treatment with GDC-0980 and/or GDC-0973, and at baseline untreated (Figure 10, Supplementary Figure S7, Supplementary Figure S8, Supplementary Figure S9, Supplementary Figure S10). pERK1/2 (Thr202/Ty204) expression could not be accurately determined due to noise from the adjacent positive control, however pERK expression should be reduced in response to GDC-0973 treatment. As such, this protein was further investigated by use of an MSD array, which contained spots for total ERK and pERK (Thr202/Tyr204, Thr185/Tyr187), allowing quantification of percentage ERK phosphorylation in response to the drug. Here, ERK phosphorylation was shown to be significantly reduced in response to both GDC-0973 treatment alone, and the combination of the two drugs, for all three cell lines (Figure 11).

pSTAT3 (Tyr705) expression was similar across all cell lines. GDC-0980 treatment both alone and in combination with GDC-0973 led to significantly decreased expression of pAKT (Thr308) in H1975 cells. GDC-0980 treatment led to significantly reduced expression of pAKT (Ser473) in H460, A549 and H1975 cells, with the effect being largest in H1975 cells. In H1975 cells, GDC-0973 treatment led to increased expression of pAKT (Ser473), but combined treatment with both inhibitors still led to a significant reduction in expression of the protein. Expression of pAMPKα (Thr172) was similar across all treatments in all cell lines. GDC-0980 treatment induced a reduction in pS6RP (Ser235/236) expression in H460, A549 and H1975 cells. As with pAKT (Ser473), this effect was largest in H1975 cells. GDC-0973 treatment also led to reduced expression of pS6RP (Ser235/236) in H460 and A549 cells. GDC-0980 induced a reduction in pmTOR (Ser2448) expression in H460, A549 and H1975 cells. GDC-0973 treatment also induced a reduction in expression of pmTOR (Ser2448) in H460 cells. GDC-0980 treatment (with or without GDC-0973) led to reduced expression of pHSP27 (Ser78) in all three cell lines. Treatment with GDC-0973 alone led to reduced expression of pHSP27 (Ser78) in H460 and A549 cells but not H1975 cells. GDC-0980 treatment (with or without GDC-0973) led to reduced expression of pBAD (Ser112) in all three cell lines. Treatment with GDC-0973 alone led to reduced expression of pBAD (Ser112) in H460 cells but not A549 or H1975 cells. GDC-0980 treatment (with or without GDC-0973) led to reduced expression of pp70S6K (Thr389) in H460, A549 and H1975 cells. Treatment with GDC-0973 alone led to reduced expression of pp70S6K (Thr389) in H460 cells but not A549 or H1975 cells. pp70S6K is a downstream mediator of both the PI3K and MEK pathways, which can be activated by either pathway. As such it would be hoped that the co-targeted inhibition approach would lead to a reduction in expression of this protein compared with treatment with either inhibitor alone. Here we saw that this was the case for H460 cells, but not either of the other two cell lines. GDC-0980 treatment (with or without GDC-0973) led to reduced expression of pPRAS40 (Thr246) in all three cell lines. GDC-0980 treatment alone induced a reduction in pp53 (Ser15) expression in all three cell lines, and the combination of the two drugs induced a reduction in expression of the protein in H460 and A549 cells but not H1975 cells. GDC-0973 treatment also induced a reduction in expression of pp53 (Ser15) in A549 cells. pp38 (Thr180/Tyr182) expression was similar across all treatments in all cell lines. GDC-0980 treatment (with or without GDC-0973) led to reduced expression of pSAPK/JNK (Thr183/Tyr185) in H460, A549 and H1975 cells. GDC-0980 treatment (with or without GDC-0973) led to reduced expression of PARP (Asp214 cleavage) in H460 cells, but not in the other two cell lines. Caspase-3 (Asp175 cleavage) expression was similar across all treatments in all cell lines. GDC-0980 treatment alone led to reduced expression of pGSK-3β (Ser9) in H460 and H1975 cells, and the mix of the two drugs induced a reduction of expression of the protein in H460, A549 and H1975 cells. GDC-073 treatment alone also led to reduced expression of pGSK-3β (Ser9) in H460 cells.

## DISCUSSION

### *PIK3CA* mutation frequency in an Irish NSCLC cohort

*PIK3CA* mutation status is known to be associated with PI3K pathway activation, which in turn is known to be involved in carcinogenesis. The PI3K pathway can be activated by a number of other molecular alterations, most notably activation of AKT, mTOR or loss of PTEN. Multiple studies have investigated the value of each of these PI3K pathway alterations as prognostic indicators, with varying findings [6]. This study investigated the frequency of PIK3CA mutations in an Irish cohort of 174 patients as part of a larger study being carried out by the European Thoracic Oncology Platform (ETOP) on a total of 2709 patients. PIK3CA was mutated in 5.26% of adenocarcinoma patients and 15.87% of squamous cell carcinoma patients from the Irish cohort and 4.6% of total ETOP cases [25]. A recent pan-PI3K inhibitor clinical trial found that PIK3CA mutation status is not an ideal method of stratifying patients for PI3K pathway targeted therapy [9]. As such, expression of key PI3K pathway phosphoproteins was profiled in 15 matched tumour and normal fresh tissue samples. Both pmTOR and pS6RP were found to be significantly more highly expressed in tumour than normal tissue, and phosphoprotein expression did not correlate with PIK3CA mutation status here. As such, we hypothesize that downstream phosphoprotein expression profiling could be a preferable method of identifying tumours with elevated PI3K pathway activation compared with PIK3CA mutation identification alone, particularly in key proteins such as mTOR and S6RP, both of which can be activated by multiple redundancies and cross-talk with other pathways including the MEK pathway.

### *PIK3CA* mutation status and PI3K pathway activation *in vitro*

Three cell lines were be used throughout this project in order to investigate the efficacy of different strategies for targeting the PI3K in NSCLC. These three cell lines were initially characterised for PI3K pathway mutation status and activation, which could then be related to the results obtained previously from NSCLC patient samples.

H460 cells have mutations in *PIK3CA* and KRAS, A549 cells have wild-type *PIK3CA* but mutated *KRAS* and *IDH1* and H1975 cells have mutated *PIK3CA, PIK3R1, CDKN2A and EGFR* but wild-type *KRAS*. *PIK3CA* gene expression was similar across the panel of cell lines (if slightly higher in H1975 cells). Expression of pAKT (Ser473), was significantly higher in H1975 cells than the other two cell lines, which could be attributed to the two PI3K pathway mutations present in this cell line, and the fact that the cell line does not have a mutation in KRAS, implying that PI3K could potentially be the driver oncogene in this cell line.

### Response to PI3K pathway inhibition in *PIK3CA* mutant versus *PIK3CA* wild-type NSCLC cell lines

It has been hypothesized that *PIK3CA* mutation status may serve as a predictive biomarker of response to PI3K targeted therapy. However, the correlation between *PIK3CA* mutation status and response to PI3K targeted therapy is controversial, with several other potential biomarkers including PTEN loss being put forward as alternatives [14]. Tumours that bear wild-type *PIK3CA* may also respond to PI3K targeted therapy, as we know that the pathway can be activated due to other aberrations such as *PTEN* loss, *AKT* or *MTOR* mutation.

While alterations in *PIK3CA* may act as driver mutations in some tumours, they could also act as passenger mutations in others. In the former case, constitutive activation of PI3K signalling occurs and the tumour is expected to be addicted to the pathway. As such, the tumour would respond well to PI3K targeted therapy. In the latter case, the *PIK3CA* mutation may not be driving tumour progression or maintenance, and as such either PI3K targeted therapy will be less effective, or drug resistance will develop more quickly as the tumour uses the co-existing driver mutation to overcome the effects of the drug.

Here we set out to investigate the effects of targeting PI3K in our panel of *PIK3CA* mutant and wild-type NSCLC cell lines. H1975 cells, which have a *PIK3CA* mutation as well as mutations in *PIK3R1, EGFR* and *CDKN2A* and no mutation in *KRAS*, were the most sensitive cell line to both PI3K pathway inhibitors, GDC-0941 (first generation, pan PI3K inhibitor) and GDC-0980 (second generation, dual PI3K-mTOR inhibitor), suggesting that *PIK3CA* may act as a driver mutation here. A549 cells, which bear wild-type *PIK3CA* but have a mutation in *KRAS* and *IDH1*, were the least sensitive to both GDC-0941 and GDC-0980, implying that the cell line does not rely on PI3K signalling. Due to the mutation in *KRAS* that is present in this cell line, it can be hypothesized that the cells are addicted to *KRAS* signalling and as such are not greatly affected by inhibition of PI3K. Finally, H460 cells were less sensitive to PI3K inhibition by GDC-0941 and GDC-0980 than H1975 cells, despite the fact that H460 cells bear a mutation in *PIK3CA*. Here, we hypothesize that *PIK3CA* is acting as a passenger mutation while the co-existing mutation in *KRAS* is driving the cell line's oncogenic properties. Both GDC-0941 and GDC-0980 do still induce anti-proliferative effects here, albeit to a lesser degree than in H1975 cells.

Based on these results we would advocate further research into the relevance of KRAS mutation status to PI3K/MEK pathway inhibition. In the future a full mutational profile of all patients that are being considered for PI3K targeted therapy should be carried out, as even in this small study differences can be seen in the response of different molecular subtypes to this form of therapy.

### Pan-PI3K inhibition versus pan-PI3K-mTOR dual inhibition in a panel of NSCLC cell lines

First generation PI3K pathway targeted drugs such as GDC-0941 inhibited the class I isoforms of PI3K. These drugs have had some success in the clinic, although even where patients initially responded to the drugs, resistance often developed rapidly [4, 13]. Further advances have led to the development of dual PI3K-mTOR inhibitors such as GDC-0980, which are hypothesized to have more potent anti-cancer effects due to their ability to circumvent feedback stimulation of PI3K-AKT-mTOR signalling.

Here we set out to compare the anti-proliferative effects of GDC-0941 with GDC-0980 in our panel of three NSCLC cell lines. In all cell lines, there was no statistically significant difference between the anti-proliferative effects of GDC-0941 and GDC-0980. However, the IC50 concentrations were lower for GDC-0980 than GDC-0941 in all three cell lines. Based on this data it is clear that there is no distinguishable difference between the two drugs in terms of their ability to induce a reduction in proliferation in the cell lines, however there is a trend toward GDC-0980 being slightly more effective here in this short term assay.

### MEK inhibition in a panel of NSCLC cell lines

A number of targeted inhibitors of the MEK pathway have been investigated in the laboratory and clinical settings, with particular interest in the field of NSCLC due to the high rate of *KRAS* mutations in the disease. However, results have been underwhelming due to the tendency of MEK inhibitors to induce growth arrest but not apoptosis [26, 27].

Here, the effects of MEK inhibitor GDC-0973 on viability and proliferation were assessed in H460, A549 and H1975 cell lines. GDC-0973 treatment did not induce a statistically significant effect on cell viability in any cell line. However, the drug did induce a reduction in proliferation in A549 and H1975 cells. The cell viability assay carried out here and throughout this project is a triple stain High Content Analysis assay which assesses changes in nuclear, cytoplasmic and mitochondrial staining, here in response to a drug. The nuclear stain, Höechst, is used to determine cell count, which is graphed to give an indication of any reduction in cell count in response to a drug. This is a method of assessing drug efficacy used throughout the literature, for example with regard to GDC-0941 and GDC-0980 efficacy as reported in Genentech's original research [15]. The triple stain approach allows for qualitative assessment of the effects of the drug on cell morphology and the visual identification of cell death. To confirm the presence of apoptosis, further investigation at the protein level was carried out, identifying a reduction in pHSP27, pBAD and p53 where apoptosis is present.

H460 cells were resistant to GDC-0973 treatment, with an IC50 concentration two orders of magnitude higher than in the other two cell lines, despite bearing a mutation in *KRAS*. H460 cells were seen to express multidrug resistance genes *ABCB1* and *ABCC2* more highly than the other cell lines, which could contribute to this effect and to the underwhelming response of H460 cells to GDC-0941 and GDC-0980.

### Co-targeted inhibition of the PI3K and MEK pathways in NSCLC

A number of studies have been published investigating the effects of co-targeted inhibition of the PI3K and MEK pathways. Several early phase clinical studies are also currently underway which combine these two types of therapies [28–30].

Initial results have been underwhelming with regards to the efficacy of the strategy. In certain cases, results have been shown to vary, depending on the presence of concurrent secondary mutations [31]. Here, we set out to assess the effects of co-targeted PI3K and MEK pathway inhibition in our panel of *PIK3CA* wild-type/mutant and *KRAS* wild-type/mutant cell lines. GDC-0980 (dual PI3K-mTOR inhibitor) and GDC-0973 (MEK inhibitor) combined treatment was shown to induce a significant reduction in cell viability compared to treatment with either drug alone in H460 and A549 cells, with a trend towards a similar response in H1975 cells. Further interrogation of the interaction between the two drugs identified a synergistic anti-proliferative response to the combined treatment approach in all cell lines, at doses as low as 0.1μM.

A detailed investigation of the effects of the combined treatment approach on intracellular signalling proteins was carried out using PathScan arrays in order to elucidate the mechanisms of inhibition at play in the different cell lines.

H460 cells exhibited a reduction in pAKT (Ser473), but not pAKT (Thr308) in response to GDC-0980, whereas the other *PIK3CA* mutated cell line, H1975, exhibited a significant reduction in both pAKT (Ser473) and pAKT (Thr308) in response to the drug. This could partially explain the reduced efficacy of the drug in H460 cells compared to H1975 cells. Here, H460 cells also exhibited a more modest reduction in pS6RP (Ser235/236) and pp70S6K (Thr389) than observed in H1975 cells in response to GDC-0980 treatment.

A549 cells exhibited a reduction in both pAKT (Ser473) and pAKT (Thr308) in response to GDC-0980. This cell line also exhibited significant reductions in pS6RP (Ser235/236), pmTOR (Ser2448) and pp70S6K (Thr389) in response to GDC-0980.

As such, this data does not offer an explanation as to why A549 cells are the least sensitive cell line to GDC-0980 treatment, however we hypothesize that either the high expression of a panel of cancer drug resistance genes identified in A549 cells, or the preferential signalling through KRAS due to KRAS mutation may help to reduce the efficacy of these inhibitors.

The signal for pERK on the arrays was quite low, and increased exposure times were problematic due to the proximity of the positive control. As such, ERK inhibition by the two drugs alone or in combination was further investigated using MSD protein arrays, which allowed for calculation of percentage ERK phosphorylation. Here, it was observed that GDC-0973 treatment led to a reduction in phosphorylation to ~0.2% in the three cell lines.

GDC-0973 treatment led to reduced pSTAT1 (Tyr701) expression in the wild-type *PIK3CA* cell line, A549. Treatment with the MEK inhibitor interestingly also induced an increased expression of pAKT (Ser473) in A549 and H1975 cells, implying that these cell lines may utilise pAKT or pSTAT1 signalling as bypass tracks in order to escape MEK inhibition. This effect was not seen with other PI3K pathway proteins included in these arrays, and as such it can be assumed that the classical PI3K-AKT-mTOR pathway is not activated here, but pAKT could be maintaining oncogenic signalling through other pathways.

The combined treatment approach led to a significantly greater reduction in protein expression compared to treatment with either inhibitor alone in certain cases, as follows. pp70S6K (Thr389) expression was significantly reduced under treatment with both drugs compared to treatment with either inhibitor alone in H460 cells, pSAPK/JNK (Thr183/Tyr185) expression was significantly reduced under treatment with both drugs compared to treatment with either inhibitor alone in A549 cells and pGSK-3β (Ser9) expression was significantly reduced under treatment with both drugs compared to treatment with either inhibitor alone in A549 cells. However, in many more cases, the combined treatment approach led to a significantly greater reduction in protein expression compared to one of the two drugs alone. As such, the observed synergy between the two drugs in all three cell lines is likely due to the combined effect of inhibiting several proteins in multiple pathways at the same time.

Data published in 2014 has suggested that the lack of initial success in combining PI3K and MEK inhibitors in the clinic may involve a failure to induce apoptosis via BCL-2 family proteins [27]. Here, a large panel of cell lines bearing a wide variety of mutations which closely mirrored the heterogeneity of NSCLC tumour types encountered in the clinic were treated with PI3K and MEK inhibitors in combination. The effects of the combination treatment varied widely across the panel of cell lines, typically inducing growth arrest but often not inducing sufficient cytotoxic effects. Restoration of BIM expression was shown to resensitize *KRAS*-mutant human and mouse cancer cells to combined PI3K and MEK inhibition, implying a role for targeting specific apoptotic mediators in addition to PI3K and MEK. This should be further investigated as a superior strategy for targeted therapy in NSCLC, however significant toxicity issues will need to be overcome in order to develop this as a viable strategy in the clinic. The emergence of third generation isoform specific PI3K inhibitors may aid in this endeavour, through the minimisation of off-target effects.

The synergistic interaction between GDC-0980 and GDC-0973 observed in this study is promising; however similar studies have been brought to the preclinical and clinical settings during the course of this study with mixed and often underwhelming results. These two drugs have not been investigated in combination in a clinical setting, although other dual PI3K-mTOR and MEK inhibitors have been investigated in combination in the clinic [19, 29, 30]. We would advocate that further preclinical and ultimately clinical studies are carried out to assess the viability of the GDC-0980 and GDC-0973 combination in NSCLC and potentially other solid tumours based on the synergy reported here. Previously published data has indicated a downregulation of PI3K pathway signalling in cisplatin-resistant NSCLC, where GDC-0980 was shown to induce a correspondingly weaker effect than that seen in cisplatin sensitive cells [32]. As such, the PI3K-MEK combined treatment strategy may be of particular use in the first line setting, prior to the development of cisplatin resistance.

## MATERIALS AND METHODS

### Drugs

GDC-0941, GDC-0980 and GDC-0973 were gifted under a material transfer agreement from Genentech for use in this study, and were dissolved in dimethyl sulphoxide (DMSO), aliquoted and stored at −20°C.

### Lung cancer cell lines

Three lung cancer cell lines were used during the course of this study; large cell lung carcinoma cells (H460), adenocarcinomic alveolar basal epithelial cells (A549) and adenocarcinoma cells (H1975). Cells were obtained from the European Culture and Tissue Collection (ECACC). H460 and H1975 cells were maintained in Roswell Park Memorial Institute (RPMI-1640) medium, which was supplemented with 10% (v/v) foetal bovine serum (FBS) and penicillin streptomycin (P/S - 5000 U/mL penicillin, 5000 U/mL streptomycin). A549 cells were maintained in F-12 (Ham) medium, which was supplemented with 10% (v/v) foetal bovine serum (FBS), penicillin streptomycin (P/S - 5000 U/mL penicillin, 5000 U/mL streptomycin), and 2 mM L-glutamine (in 0.85% NaCl). All three cells lines are adherent and were incubated in vented flasks at 37°C in 95% air, 5% CO_2_ humidified atmosphere (Steri-Cycle CO_2_ incubator, ThermoForma, Marietta, OH, USA). Cell lines were passaged every 48-72hrs and tested for mycoplasma once per month by the polymerase chain reaction (PCR) method [33]. Cell lines sample from before, during and after the period during which these experiments were carried out were authenticated by DNA Diagnostic Centre.

### NSCLC patient mutational profiling

As part of the European Thoracic Oncology Platform (ETOP) Lungscape project [25] which profiled a total of 2709 NSCLC patient tumours from 17 predominantly European centres, 175 Irish NSCLC tumour samples were profiled by Fluidigm Technology, using an allele-specific test covering 13 genes (150 mutations, Supplementary Table 1).

### RT^2^ profiler arrays

RT^2^ Profiler arrays (SABiosciences) were used to compare gene expression across cell line samples. Each 96 well array contains primers for 84 genes of interest along with 12 controls. RNA was isolated using the RNeasy mini kit and cDNA was synthesized using the first strand kit. The PCR reaction contained SYBR green mastermix, cDNA synthesis reaction (1μg) and molecular grade water. Arrays were cycled using the 7900HT qPCR machine (Applied Biosystems) as per manufacturer's protocol. Data was analysed using SABiosciences online software (2.6.6).

### High content analysis viability assay

Cells were triple stained (nuclear, mitochondrial and cytoskeletal stains), imaged and analysed in order to assess viability and induction of apoptosis both qualitatively and quantitatively. Cells were counted, seeded at 2000 cells per well and treated as noted for a period of 48 hrs. Cells were stained simultaneously with Höechst nuclear stain (bisbenzimide H33348, FlukaBiochemika, 1μM) and Mitotracker Red mitochondrial stain (Invitrogen, CA, USA, 0.5μM) for 30 minutes, then washed in PBS and fixed in paraformaldehyde (4%, 10 minutes) before permeablisation (0.5% Triton-X-100 in PBS). Cells were then stained with Alexa Fluor 488 Phalloidin (Invitrogen, 10μM) and imaged using the InCell Analyser 1000 (GE Lifesciences). Images were analysed using High Content Analysis (HCA) software, which quantifies multiple parameters including cell count, intensity of nuclear, mitochondrial and cytoplasmic staining, area of nucleus, mitochondria and cytoplasm, and nuclear/cytoplasmic area or intensity.

### BrdU proliferation assay

Cell proliferation was measured using a Cell Proliferation enzyme linked immunosorbent assay (ELISA), BrdU (Roche Diagnostics Ltd.). This is a colorimetric method to quantify cell proliferation based on the measurement of BrdU (5-bromo-2'-deoxyuridine - a pyrimidine analogue) incorporation (instead of thymidine) during DNA synthesis in proliferating cells. Cells were seeded at 2000 cells per well in a 96-well plate and adhered overnight (o/n). Cells were treated with appropriate drugs for 72 hours unless otherwise noted. Following treatment, 10μL of a 1:1000 dilution of BrdU labelling solution (final concentration - 10μM) was added to each well and plates incubated for 4 hours at 37°C. Following incubation, the media was removed and the cells fixed and denatured with 200μL of a fixative solution for 30 minutes at room temperature (RT). 100μL anti-BrdU-POD (mouse monoclonal antibody, peroxidase-conjugated) working solution was added to each well for 90 minutes at RT. Cells were washed three times with wash buffer and 100μL of substrate solution was added for 5-10 minutes (or until colour change was sufficient for photometric detection). 25μL 1 mM H_2_SO_4_ was added to each well to stop the reaction. Absorbance was measured on a Vesamax tunable microplate reader (Molecular Devices) at 450nm with a reference wavelength set to 690nm.

### PathScan arrays

PathScan arrays (Cell Signalling Technologies) are slide based antibody arrays which were used to profile expression of 18 phosphorylated or cleaved proteins across a panel of untreated and drug treated cell line samples. Capture antibodies are spotted in duplicate onto nitrocellulose coated glass slides, with 16 arrays per slide allowing for simultaneous comparison of protein expression across 15 samples and a control. Cell line protein samples were prepared and quantified as per manufacturer's protocol. NSCLC patient fresh tissue samples were stabilised immediately using ALL protect (Qiagen) and then protein was isolated as per CST guidelines. 100μL blocking buffer was added to each well and incubated at RT for 15 minutes on a shaker. Cell lysates are diluted to 1mg/mL and 75μL of this working solution is added per well and incubated for overnight at 4°C on a shaker. Four 5 minute washes were carried out using 1X array wash buffer, and 75μL detection antibody cocktail was added. The slide was incubated at RT for 1 hour on a shaker, then four further 5 minute washes were carried out. 75μL HRP-linked Strepdavidin was added to each well and incubated for 30 minutes at RT on a shaker. Four further 5 minute washes were carried out prior to incubation in LumiGlo/peroxide mix (chemiluminescent reagent) for the duration of slide imaging using a Biospectrum Imaging System (Ultra Violet Products). Densitometry analysis was carried out using ImageJ.

## SUPPLEMENTARY FIGURES AND TABLE




