# Supplementary material for: In pursuit of synergy: An investigation of the PI3K/mTOR/MEK co-targeted inhibition strategy in NSCLC
Source: Oncotarget. 2016 Oct 19;7(48):79526–43. doi: 10.18632/oncotarget.12755 (PMC5346733; doi:10.18632/oncotarget.12755)
Supplement: Supplementary file 1 [file oncotarget-07-79526-s001.pdf]

In pursuit of synergy: An investigation of the PI3K/mTOR/MEK co-targeted inhibition strategy in NSCLC

SUPPLEMENTARY FIGURES AND TABLE

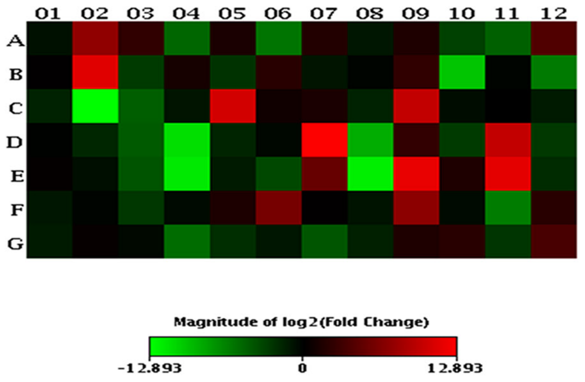

Layout	01	02	03	04	05	06	07	08	09	10	11	12
A	Akt1<br>-1.74<br>OKAY	Akt1s1<br>165.10<br>A	Akt2<br>5.42<br>OKAY	Akt3<br>-36.56<br>OKAY	Cab39<br>2.40<br>OKAY	Cab39l<br>-57.08<br>OKAY	Cdc42<br>3.24<br>OKAY	Chuk<br>-2.15<br>OKAY	Ddit4<br>2.89<br>OKAY	Ddit4l<br>-9.14<br>OKAY	Deptor<br>-28.38<br>OKAY	Eif4b<br>16.99<br>OKAY
B	Eif4e<br>1.07<br>OKAY	Eif4ebp1<br>2706.94<br>A	Eif4ebp2<br>-7.73<br>OKAY	Fkbp1a<br>2.16<br>OKAY	Fkbp8<br>-5.83<br>OKAY	Gsk3b<br>3.93<br>OKAY	Hif1a<br>-2.02<br>OKAY	Hras1<br>-1.12<br>OKAY	Hspa4<br>5.44<br>OKAY	Igf1<br>-1007.08<br>A	Igfbp3<br>-1.16<br>OKAY	Ikbkb<br>-73.96<br>OKAY
C	Ilk<br>-3.27<br>OKAY	Ins2<br>-7215.66<br>A	Insr<br>-26.92<br>OKAY	Irs1<br>-2.05<br>OKAY	Mapk1<br>1717.71<br>A	Mapk3<br>1.80<br>OKAY	Mapkap1<br>2.41<br>OKAY	Mlst8<br>-3.21<br>OKAY	Mtor<br>951.67<br>A	Myo1c<br>-1.55<br>OKAY	Pdpk1<br>1.01<br>OKAY	Pik3c3<br>-2.46<br>OKAY
D	Pik3ca<br>-1.04<br>OKAY	Pik3cb<br>-3.91<br>OKAY	Pik3cd<br>-23.61<br>OKAY	Pik3cg<br>-2536.82<br>A	Pld1<br>-3.63<br>OKAY	Pld2<br>-1.26<br>OKAY	Ppp2ca<br>7606.65<br>A	Ppp2r2b<br>-451.23<br>A	Ppp2r4<br>5.93<br>OKAY	Prkaa1<br>-7.40<br>OKAY	Prkaa2<br>984.27<br>A	Prkab1<br>-7.10<br>OKAY
E	Prkab2<br>1.15<br>OKAY	Prkag1<br>-1.69<br>OKAY	Prkag2<br>-19.66<br>OKAY	Prkag3<br>-3564.03<br>A	Prkca<br>-2.56<br>OKAY	Prkcb<br>-12.16<br>OKAY	Prkcg<br>34.90<br>A	Prkce<br>-4565.00<br>A	Pten<br>3634.11<br>A	Rheb<br>2.82<br>OKAY	Rhoa<br>3078.48<br>A	Rictor<br>-4.30<br>OKAY
F	Rps6<br>-2.10<br>OKAY	Rps6ka1<br>-1.17<br>OKAY	Rps6ka2<br>-6.50<br>OKAY	Rps6ka5<br>-1.39<br>OKAY	Rps6kb1<br>2.70<br>OKAY	Rps6kb2<br>63.40<br>A	Rptor<br>1.04<br>OKAY	Rraga<br>-1.94<br>OKAY	Rragb<br>140.59<br>A	Rragc<br>-1.46<br>OKAY	Rragd<br>-77.26<br>OKAY	Sgk1<br>4.10<br>OKAY
G	Stk11<br>-2.42<br>OKAY	Stradb<br>1.20<br>OKAY	Telo2<br>-1.29<br>OKAY	Trp53<br>-43.42<br>OKAY	Tsc1<br>-4.86<br>OKAY	Tsc2<br>-2.15<br>OKAY	Ulk1<br>-20.97<br>OKAY	Ulk2<br>-3.06<br>OKAY	Vegfa<br>2.93<br>OKAY	Vegfb<br>4.02<br>OKAY	Vegfc<br>-6.22<br>OKAY	Ywhaq<br>12.32<br>OKAY

Supplementary Figure S1: mTOR pathway mRNA up/down-regulation in H460 cells compared to and A549s. RNA was extracted from H460 and A549 cells. cDNA was synthesized, which was then added to mTOR pathway RT^2 Profiler Arrays. H460 gene expression was compared to A549 gene expression. A heat map was constructed using SABiosciences online software, with fold changes shown below, where 'A' refers to genes which amplified late (>30 cycles) in either sample, 'B' refers to genes which amplified late (>30 cycles) in both samples, and 'C' refers to samples where no expression was detected in either sample. 27 genes were upregulated >2 fold and 39 genes were downregulated >2 fold) in H460 cells compared with A549 cells.

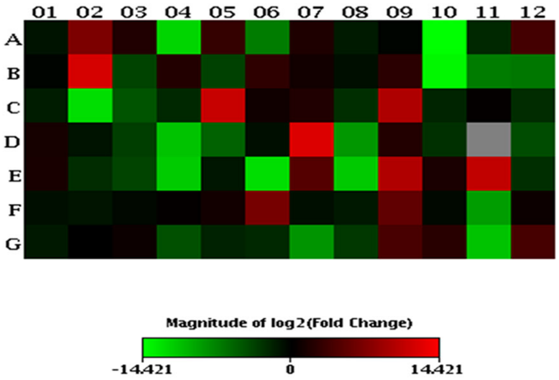

Layout	01	02	03	04	05	06	07	08	09	10	11	12
**A**	Akt1<br>**-2.03**<br>OKAY	Akt1s1<br>**130.99**<br>A	Akt2<br>**3.48**<br>OKAY	Akt3<br>**-4396.99**<br>A	Cab39<br>**7.96**<br>OKAY	Cab39l<br>**-124.22**<br>OKAY	Cdc42<br>**3.22**<br>OKAY	Chuk<br>**-2.69**<br>OKAY	Ddit4<br>**-1.13**<br>OKAY	Ddit4l<br>**-21929.11**<br>A	Deptor<br>**-4.72**<br>OKAY	Eif4b<br>**14.73**<br>OKAY
**B**	Eif4e<br>**-1.21**<br>OKAY	Eif4ebp1<br>**4548.62**<br>A	Eif4ebp2<br>**-12.88**<br>OKAY	Fkbp1a<br>**3.91**<br>OKAY	Fkbp8<br>**-12.14**<br>OKAY	Gsk3b<br>**6.12**<br>OKAY	Hif1a<br>**2.07**<br>OKAY	Hras1<br>**-1.68**<br>OKAY	Hspa4<br>**5.29**<br>OKAY	Igf1<br>**-16289.27**<br>A	Igfbp3<br>**-126.95**<br>OKAY	Ikbkb<br>**-111.27**<br>OKAY
**C**	Ilk<br>**-3.11**<br>OKAY	Ins2<br>**-6120.15**<br>A	Insr<br>**-27.85**<br>OKAY	Irs1<br>**-4.71**<br>OKAY	Mapk1<br>**2447.82**<br>A	Mapk3<br>**1.82**<br>OKAY	Mapkap1<br>**3.56**<br>OKAY	Mlst8<br>**-5.85**<br>OKAY	Mtor<br>**1015.72**<br>A	Myo1c<br>**-4.23**<br>OKAY	Pdpk1<br>**1.21**<br>OKAY	Pik3c3<br>**-5.41**<br>OKAY
**D**	Pik3ca<br>**2.33**<br>OKAY	Pik3cb<br>**-2.07**<br>OKAY	Pik3cd<br>**-11.48**<br>OKAY	Pik3cg<br>**-2151.67**<br>A	Pld1<br>**-45.77**<br>OKAY	Pld2<br>**-1.77**<br>OKAY	Ppp2ca<br>**5895.18**<br>A	Ppp2r2b<br>**-382.72**<br>A	Ppp2r4<br>**3.85**<br>OKAY	Prkaa1<br>**-6.59**<br>OKAY	Prkaa2<br>**-2.60**<br>C	Prkab1<br>**-19.54**<br>OKAY
**E**	Prkab2<br>**2.58**<br>OKAY	Prkag1<br>**-5.82**<br>OKAY	Prkag2<br>**-14.47**<br>OKAY	Prkag3<br>**-3022.93**<br>A	Prkca<br>**-2.36**<br>OKAY	Prkcb<br>**-5913.82**<br>A	Prkcg<br>**27.31**<br>A	Prkce<br>**-2757.04**<br>A	Pten<br>**1055.37**<br>A	Rheb<br>**2.85**<br>OKAY	Rhoa<br>**2040.93**<br>A	Rictor<br>**-5.98**<br>OKAY
**F**	Rps6<br>**-1.69**<br>OKAY	Rps6ka1<br>**-2.26**<br>OKAY	Rps6ka2<br>**-1.36**<br>OKAY	Rps6ka5<br>**1.18**<br>OKAY	Rps6kb1<br>**1.98**<br>OKAY	Rps6kb2<br>**98.95**<br>A	Rptor<br>**-1.79**<br>OKAY	Rraga<br>**-2.50**<br>OKAY	Rragb<br>**43.38**<br>A	Rragc<br>**-1.37**<br>OKAY	Rragd<br>**-500.88**<br>A	Sgk1<br>**1.63**<br>OKAY
**G**	Stk11<br>**-2.53**<br>OKAY	Stradb<br>**-1.04**<br>OKAY	Telo2<br>**1.56**<br>OKAY	Trp53<br>**-23.51**<br>OKAY	Tsc1<br>**-3.91**<br>OKAY	Tsc2<br>**-4.88**<br>OKAY	Ulk1<br>**-358.85**<br>OKAY	Ulk2<br>**-8.76**<br>OKAY	Vegfa<br>**16.27**<br>OKAY	Vegfb<br>**4.89**<br>OKAY	Vegfc<br>**-2130.25**<br>A	Ywhaq<br>**15.31**<br>OKAY

Supplementary Figure S2: mTOR pathway mRNA up/down-regulation in H1975 cells compared to and A549 cells. RNA was extracted from H1975 and A549 cells. cDNA was synthesized, which was then added to mTOR pathway RT^2 Profiler Arrays. H1975 gene expression was compared to A549 gene expression. A heat map was constructed using SABiosciences online software, with fold changes shown below, where ‘A’ refers to genes which amplified late (>30 cycles) in either sample, ‘B’ refers to genes which amplified late (>30 cycles) in both samples, and ‘C’ refers to samples where no expression was detected in either sample. 26 genes were upregulated >2 fold and 41 genes were downregulated >2 fold in H1975 cells compared with A549 cells.

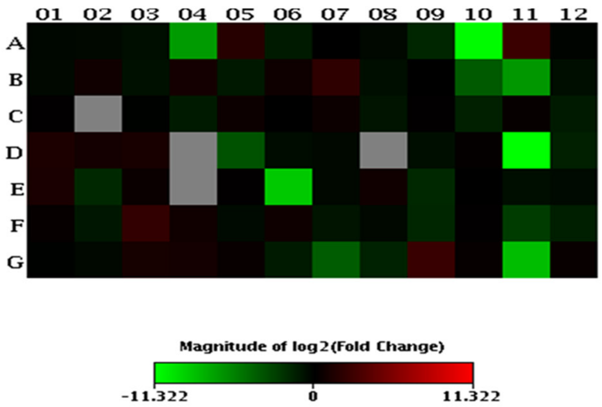

Layout	01	02	03	04	05	06	07	08	09	10	11	12
**A**	Akt1<br>**-1.17**<br>OKAY	Akt1s1<br>**-1.26**<br>OKAY	Akt2<br>**-1.56**<br>OKAY	Akt3<br>**-120.27**<br>A	Cab39<br>**3.31**<br>OKAY	Cab39l<br>**-2.18**<br>OKAY	Cdc42<br>**-1.01**<br>OKAY	Chuk<br>**-1.25**<br>OKAY	Ddit4<br>**-3.26**<br>OKAY	Ddit4l<br>**-2399.85**<br>A	Deptor<br>**6.01**<br>OKAY	Eif4b<br>**-1.15**<br>OKAY
**B**	Eif4e<br>**-1.29**<br>OKAY	Eif4ebp1<br>**1.68**<br>OKAY	Eif4ebp2<br>**-1.67**<br>OKAY	Fkbp1a<br>**1.81**<br>OKAY	Fkbp8<br>**-2.08**<br>OKAY	Gsk3b<br>**1.56**<br>OKAY	Hif1a<br>**4.17**<br>OKAY	Hras1<br>**-1.51**<br>OKAY	Hspa4<br>**-1.03**<br>OKAY	Igf1<br>**-16.17**<br>B	Igfbp3<br>**-109.24**<br>OKAY	Ikbkb<br>**-1.50**<br>OKAY
**C**	Ilk<br>**1.05**<br>OKAY	Ins2<br>**1.18**<br>C	Insr<br>**-1.03**<br>OKAY	Irs1<br>**-2.29**<br>OKAY	Mapk1<br>**1.43**<br>OKAY	Mapk3<br>**1.01**<br>OKAY	Mapkap1<br>**1.48**<br>OKAY	Mlst8<br>**-1.83**<br>OKAY	Mtor<br>**1.07**<br>OKAY	Myo1c<br>**-2.73**<br>OKAY	Pdpk1<br>**1.20**<br>OKAY	Pik3c3<br>**-2.20**<br>OKAY
**D**	Pik3ca<br>**2.44**<br>OKAY	Pik3cb<br>**1.89**<br>OKAY	Pik3cd<br>**2.06**<br>OKAY	Pik3cg<br>**1.18**<br>C	Pld1<br>**-12.61**<br>OKAY	Pld2<br>**-1.41**<br>OKAY	Ppp2ca<br>**-1.29**<br>OKAY	Ppp2r2b<br>**1.18**<br>C	Ppp2r4<br>**-1.54**<br>OKAY	Prkaa1<br>**1.12**<br>OKAY	Prkaa2<br>**-2560.61**<br>A	Prkab1<br>**-2.75**<br>OKAY
**E**	Prkab2<br>**2.24**<br>OKAY	Prkag1<br>**-3.45**<br>OKAY	Prkag2<br>**1.36**<br>OKAY	Prkag3<br>**1.18**<br>C	Prkca<br>**1.08**<br>OKAY	Prkcb<br>**-486.53**<br>A	Prkcg<br>**-1.28**<br>OKAY	Prkce<br>**1.66**<br>B	Pten<br>**-3.44**<br>OKAY	Rheb<br>**1.01**<br>OKAY	Rhoa<br>**-1.51**<br>OKAY	Rictor<br>**-1.39**<br>OKAY
**F**	Rps6<br>**1.24**<br>OKAY	Rps6ka1<br>**-1.93**<br>OKAY	Rps6ka2<br>**4.78**<br>OKAY	Rps6ka5<br>**1.64**<br>OKAY	Rps6kb1<br>**-1.37**<br>OKAY	Rps6kb2<br>**1.56**<br>OKAY	Rptor<br>**-1.86**<br>OKAY	Rraga<br>**-1.29**<br>OKAY	Rragb<br>**-3.24**<br>OKAY	Rragc<br>**1.07**<br>OKAY	Rragd<br>**-6.48**<br>A	Sgk1<br>**-2.51**<br>OKAY
**G**	Stk11<br>**-1.05**<br>OKAY	Stradb<br>**-1.25**<br>OKAY	Telo2<br>**2.02**<br>OKAY	Trp53<br>**1.85**<br>OKAY	Tsc1<br>**1.24**<br>OKAY	Tsc2<br>**-2.27**<br>OKAY	Ulk1<br>**-17.11**<br>OKAY	Ulk2<br>**-2.86**<br>OKAY	Vegfa<br>**5.56**<br>OKAY	Vegfb<br>**1.22**<br>OKAY	Vegfc<br>**-342.61**<br>A	Ywhaq<br>**1.24**<br>OKAY

Supplementary Figure S3: mTOR pathway mRNA up/down-regulation in H1975 cells compared to and H460 cells. RNA was extracted from H1975 and H460 cells. cDNA was synthesized, which was then added to mTOR pathway RT^2 Profiler Arrays. H1975 gene expression was compared to H460 gene expression. A heat map was constructed using SABiosciences online software, with fold changes shown below, where ‘A’ refers to genes which amplified late (>30 cycles) in either sample, ‘B’ refers to genes which amplified late (>30 cycles) in both samples, and ‘C’ refers to samples where no expression was detected in either sample. 9 genes were upregulated > 2 fold and 23 genes were downregulated in H1975 cells compared with H460 cells.

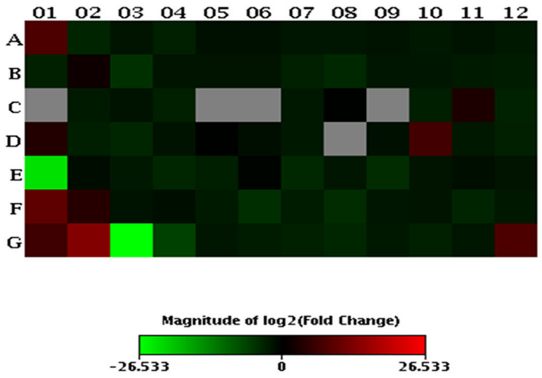

Layout	01	02	03	04	05	06	07	08	09	10	11	12
**A**	ABCB1<br>**250.43**<br>A	ABCC1<br>**-13.71**<br>OKAY	ABCC2<br>**-4.42**<br>OKAY	ABCC3<br>**-9.53**<br>OKAY	ABCC5<br>**-3.29**<br>OKAY	ABCG2<br>**-2.99**<br>OKAY	AHR<br>**-5.12**<br>OKAY	AP1S1<br>**-4.66**<br>OKAY	APC<br>**-3.62**<br>OKAY	AR<br>**-5.42**<br>OKAY	ARNT<br>**-3.60**<br>OKAY	ATM<br>**-5.40**<br>OKAY
**B**	BAX<br>**-9.59**<br>OKAY	BCL2<br>**2.71**<br>OKAY	BCL2L1<br>**-30.74**<br>OKAY	BLMH<br>**-4.37**<br>OKAY	BRCA1<br>**-4.45**<br>OKAY	BRCA2<br>**-4.22**<br>OKAY	CCND1<br>**-9.59**<br>OKAY	CCNE1<br>**-16.98**<br>OKAY	CDK2<br>**-4.75**<br>OKAY	CDK4<br>**-4.72**<br>OKAY	CDKN1A<br>**-6.90**<br>OKAY	CDKN1B<br>**-7.24**<br>OKAY
**C**	CDKN2A<br>**-3.15**<br>C	CDKN2D<br>**-6.79**<br>OKAY	CLPTM1L<br>**-3.49**<br>OKAY	CYP1A1<br>**-10.40**<br>A	CYP1A2<br>**-3.15**<br>C	CYP2B6<br>**-3.15**<br>C	CYP2C19<br>**-5.38**<br>B	CYP2C8<br>**-1.08**<br>B	CYP2C9<br>**-3.15**<br>C	CYP2D6<br>**-8.62**<br>OKAY	CYP2E1<br>**7.26**<br>A	CYP3A4<br>**-11.60**<br>B
**D**	CYP3A5<br>**12.36**<br>A	DHFR<br>**-8.97**<br>OKAY	EGFR<br>**-16.57**<br>OKAY	ELK1<br>**-3.58**<br>OKAY	EPHX1<br>**-1.22**<br>OKAY	ERBB2<br>**-2.86**<br>OKAY	ERBB3<br>**-5.27**<br>A	ERBB4<br>**-3.15**<br>C	ERCC3<br>**-3.19**<br>OKAY	ESR1<br>**122.26**<br>A	ESR2<br>**-5.98**<br>B	FGF2<br>**-9.46**<br>OKAY
**E**	FOS<br>**-12345286.65**<br>OKAY	GSK3A<br>**-2.50**<br>OKAY	GSTP1<br>**-5.48**<br>OKAY	HIF1A<br>**-15.68**<br>OKAY	IGF1R<br>**-8.90**<br>OKAY	IGF2R<br>**-1.36**<br>OKAY	MET<br>**-18.83**<br>OKAY	MSH2<br>**-5.18**<br>OKAY	MVP<br>**-23.78**<br>OKAY	MYC<br>**-4.01**<br>OKAY	NAT2<br>**-2.76**<br>A	NFKB1<br>**-4.47**<br>OKAY
**F**	NFKB2<br>**865.93**<br>A	NFKBIB<br>**14.96**<br>A	NFKBIE<br>**-3.90**<br>OKAY	PPARA<br>**-2.87**<br>OKAY	PPARD<br>**-6.74**<br>OKAY	PPARG<br>**-26.55**<br>OKAY	RARA<br>**-7.80**<br>OKAY	RARB<br>**-25.22**<br>OKAY	RARG<br>**-5.08**<br>OKAY	RB1<br>**-4.49**<br>OKAY	RELB<br>**-12.72**<br>OKAY	RXRA<br>**-5.26**<br>OKAY
**G**	RXRB<br>**89.25**<br>A	SOD1<br>**14609.22**<br>A	SULT1E1<br>**-97118311.28**<br>A	TNFRSF11A<br>**-102.30**<br>A	TOP1<br>**-4.56**<br>OKAY	TOP2A<br>**-7.87**<br>OKAY	TOP2B<br>**-10.02**<br>OKAY	TP53<br>**-14.59**<br>OKAY	TPMT<br>**-5.55**<br>OKAY	UGCG<br>**-10.74**<br>OKAY	XPA<br>**-5.20**<br>OKAY	XPC<br>**245.80**<br>OKAY

Supplementary Figure S4: Cancer drug resistance mRNA up/down-regulation in H460 cells compared to A549 cells. RNA was extracted from H460 and A549 cells. cDNA was synthesized, which was then added to cancer drug resistance RT^2 Profiler Arrays. H460 gene expression was compared to A549 gene expression. A heat map was constructed using SABiosciences online software, with fold changes shown below, where 'A' refers to genes which amplified late (>30 cycles) in either sample, 'B' refers to genes which amplified late (>30 cycles) in both samples, and 'C' refers to samples where no expression was detected in either sample. 9 genes were upregulated > 2 fold and 65 genes were downregulated > 2 fold in H460 cells compared with A549 cells.

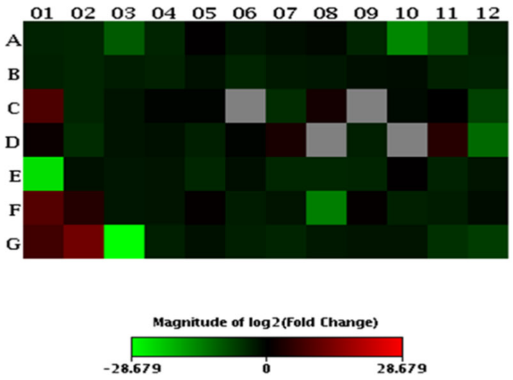

Layout	01	02	03	04	05	06	07	08	09	10	11	12
**A**	ABCB1<br>**-14.11**<br>B	ABCC1<br>**-17.49**<br>OKAY	ABCC2<br>**-1049.74**<br>OKAY	ABCC3<br>**-16.09**<br>OKAY	ABCC5<br>**1.10**<br>OKAY	ABCG2<br>**-5.77**<br>OKAY	AHR<br>**-2.80**<br>OKAY	AP1S1<br>**-1.88**<br>OKAY	APC<br>**-17.80**<br>OKAY	AR<br>**-39194.01**<br>A	ARNT<br>**-712.26**<br>A	ATM<br>**-10.01**<br>OKAY
**B**	BAX<br>**-10.73**<br>OKAY	BCL2<br>**-16.90**<br>OKAY	BCL2L1<br>**-8.68**<br>OKAY	BLMH<br>**-12.84**<br>OKAY	BRCA1<br>**-3.70**<br>OKAY	BRCA2<br>**-17.38**<br>OKAY	CCND1<br>**-6.07**<br>OKAY	CCNE1<br>**-5.32**<br>OKAY	CDK2<br>**-2.81**<br>OKAY	CDK4<br>**-2.58**<br>OKAY	CDKN1A<br>**-11.61**<br>OKAY	CDKN1B<br>**-13.50**<br>OKAY
**C**	CDKN2A<br>**362.66**<br>A	CDKN2D<br>**-17.64**<br>OKAY	CLPTM1L<br>**-4.16**<br>OKAY	CYP1A1<br>**-1.39**<br>OKAY	CYP1A2<br>**-1.47**<br>B	CYP2B6<br>**-18.17**<br>C	CYP2C19<br>**-31.04**<br>B	CYP2C8<br>**4.75**<br>A	CYP2C9<br>**-18.17**<br>C	CYP2D6<br>**-2.14**<br>OKAY	CYP2E1<br>**-1.05**<br>A	CYP3A4<br>**-137.74**<br>B
**D**	CYP3A5<br>**2.07**<br>A	DHFR<br>**-27.37**<br>OKAY	EGFR<br>**-4.05**<br>OKAY	ELK1<br>**-3.57**<br>OKAY	EPHX1<br>**-11.00**<br>OKAY	ERBB2<br>**-1.46**<br>OKAY	ERBB3<br>**6.17**<br>OKAY	ERBB4<br>**-18.17**<br>C	ERCC3<br>**-9.76**<br>OKAY	ESR1<br>**-18.17**<br>C	ESR2<br>**19.92**<br>A	FGF2<br>**-3952.55**<br>OKAY
**E**	FOS<br>**-35994733.47**<br>OKAY	GSK3A<br>**-3.63**<br>OKAY	GSTP1<br>**-5.96**<br>OKAY	HIF1A<br>**-4.55**<br>OKAY	IGF1R<br>**-18.37**<br>OKAY	IGF2R<br>**-2.88**<br>OKAY	MET<br>**-19.18**<br>OKAY	MSH2<br>**-19.16**<br>OKAY	MVP<br>**-17.41**<br>OKAY	MYC<br>**1.10**<br>OKAY	NAT2<br>**-15.00**<br>A	NFKB1<br>**-4.68**<br>OKAY
**F**	NFKB2<br>**683.36**<br>A	NFKBIB<br>**15.74**<br>A	NFKBIE<br>**-5.80**<br>OKAY	PPARA<br>**-4.74**<br>OKAY	PPARD<br>**1.28**<br>OKAY	PPARG<br>**-8.63**<br>OKAY	RARA<br>**-4.72**<br>OKAY	RARB<br>**-17159.69**<br>A	RARG<br>**1.33**<br>OKAY	RB1<br>**-12.08**<br>OKAY	RELB<br>**-8.24**<br>OKAY	RXRA<br>**-2.58**<br>OKAY
**G**	RXRB<br>**145.52**<br>A	SOD1<br>**6595.14**<br>A	SULT1E1<br>**-429817058.38**<br>A	TNFRSF11A<br>**-9.64**<br>OKAY	TOP1<br>**-3.38**<br>OKAY	TOP2A<br>**-10.49**<br>OKAY	TOP2B<br>**-17.45**<br>OKAY	TP53<br>**-6.75**<br>OKAY	TPMT<br>**-4.68**<br>OKAY	UGCG<br>**-4.66**<br>OKAY	XPA<br>**-45.43**<br>OKAY	XPC<br>**-110.60**<br>OKAY

Supplementary Figure S5: Cancer drug resistance mRNA up/down-regulation in H1975 cells compared to A549 cells. RNA was extracted from H1975 and A549 cells. cDNA was synthesized, which was then added to cancer drug resistance RT^2 Profiler Arrays. H1975 gene expression was compared to A549 gene expression. A heat map was constructed using SABiosciences online software, with fold changes shown below, where 'A' refers to genes which amplified late (>30 cycles) in either sample, 'B' refers to genes which amplified late (>30 cycles) in both samples, and 'C' refers to samples where no expression was detected in either sample. 9 genes were upregulated > 2 fold and 60 genes were downregulated > 2 fold in H1975 cells compared with A549 cells.

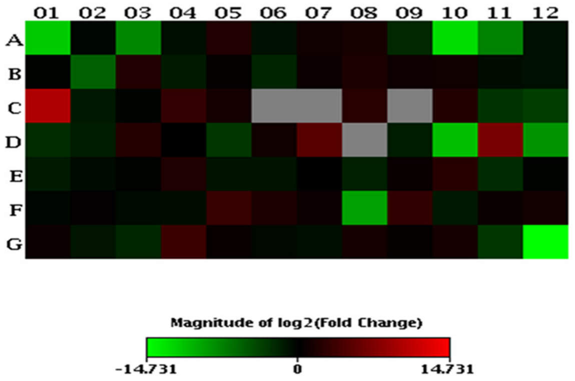

Layout	01	02	03	04	05	06	07	08	09	10	11	12
A	ABCB1<br>-3532.55<br>A	ABCC1<br>-1.28<br>OKAY	ABCC2<br>-237.48<br>OKAY	ABCC3<br>-1.69<br>OKAY	ABCC5<br>3.63<br>OKAY	ABCG2<br>-1.93<br>OKAY	AHR<br>1.83<br>OKAY	AP1S1<br>2.47<br>OKAY	APC<br>-4.92<br>OKAY	AR<br>-7231.58<br>A	ARNT<br>-197.97<br>A	ATM<br>-1.85<br>OKAY
B	BAX<br>-1.12<br>OKAY	BCL2<br>-45.81<br>OKAY	BCL2L1<br>3.54<br>OKAY	BLMH<br>-2.94<br>OKAY	BRCA1<br>1.20<br>OKAY	BRCA2<br>-4.12<br>OKAY	CCND1<br>1.58<br>OKAY	CCNE1<br>3.19<br>OKAY	CDK2<br>1.69<br>OKAY	CDK4<br>1.83<br>OKAY	CDKN1A<br>-1.68<br>OKAY	CDKN1B<br>-1.86<br>OKAY
C	CDKN2A<br>1141.35<br>A	CDKN2D<br>-2.60<br>OKAY	CLPTM1L<br>-1.19<br>OKAY	CYP1A1<br>7.49<br>A	CYP1A2<br>2.14<br>B	CYP2B6<br>-5.77<br>C	CYP2C19<br>-5.77<br>C	CYP2C8<br>5.13<br>A	CYP2C9<br>-5.77<br>C	CYP2D6<br>4.03<br>OKAY	CYP2E1<br>-7.59<br>OKAY	CYP3A4<br>-11.87<br>B
D	CYP3A5<br>-5.97<br>OKAY	DHFR<br>-3.05<br>OKAY	EGFR<br>4.09<br>OKAY	ELK1<br>1.00<br>OKAY	EPHX1<br>-9.00<br>OKAY	ERBB2<br>1.96<br>OKAY	ERBB3<br>32.54<br>A	ERBB4<br>-5.77<br>C	ERCC3<br>-3.06<br>OKAY	ESR1<br>-2221.47<br>A	ESR2<br>119.11<br>A	FGF2<br>-417.78<br>OKAY
E	FOS<br>-2.92<br>OKAY	GSK3A<br>-1.45<br>OKAY	GSTP1<br>-1.09<br>OKAY	HIF1A<br>3.44<br>OKAY	IGF1R<br>-2.06<br>OKAY	IGF2R<br>-2.12<br>OKAY	MET<br>-1.02<br>OKAY	MSH2<br>-3.70<br>OKAY	MVP<br>1.37<br>OKAY	MYC<br>4.42<br>OKAY	NAT2<br>-5.44<br>OKAY	NFKB1<br>-1.05<br>OKAY
F	NFKB2<br>-1.27<br>OKAY	NFKBIB<br>1.05<br>OKAY	NFKBIE<br>-1.49<br>OKAY	PPARA<br>-1.65<br>OKAY	PPARD<br>8.64<br>OKAY	PPARG<br>3.08<br>OKAY	RARA<br>1.65<br>OKAY	RARB<br>-680.38<br>A	RARG<br>6.73<br>OKAY	RB1<br>-2.69<br>OKAY	RELB<br>1.54<br>OKAY	RXRA<br>2.04<br>OKAY
G	RXRB<br>1.63<br>OKAY	SOD1<br>-2.22<br>OKAY	SULT1E1<br>-4.43<br>B	TNFRSF11A<br>10.62<br>A	TOP1<br>1.35<br>OKAY	TOP2A<br>-1.33<br>OKAY	TOP2B<br>-1.74<br>OKAY	TP53<br>2.16<br>OKAY	TPMT<br>1.19<br>OKAY	UGCG<br>2.31<br>OKAY	XPA<br>-8.74<br>OKAY	XPC<br>-27186.46<br>OKAY

Supplementary Figure S6: Cancer drug resistance mRNA up/down-regulation in H1975 cells compared to H460 cells. RNA was extracted from H1975 and H460 cells. cDNA was synthesized, which was then added to cancer drug resistance RT^2 Profiler Arrays. H1975 gene expression was compared to H460 gene expression. A heat map was constructed using SABiosciences online software, with fold changes shown below, where 'A' refers to genes which amplified late (>30 cycles) in either sample, 'B' refers to genes which amplified late (>30 cycles) in both samples, and 'C' refers to samples where no expression was detected in either sample. 20 genes were upregulated >2 fold and 26 genes were downregulated in H1975 cells compared with H460 **cells.**

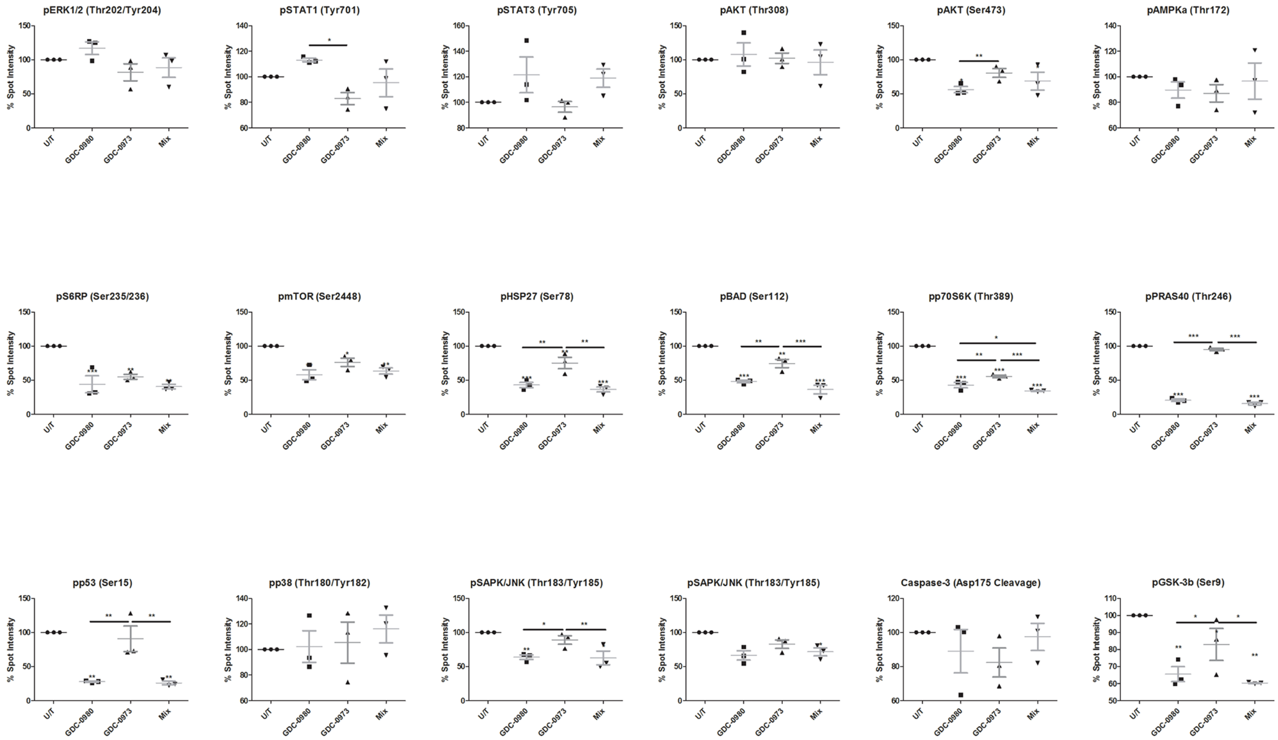

Supplementary Figure S7: Effect of GDC-0980 and/or GDC-0973 treatment on phosphoprotein expression in H460 cells. Protein was isolated from H460 cells untreated (4hrs) and treated with GDC-0980 (1µM, 4hrs), GDC-0973 (1µM, 4hrs) and a 1:1 mix of the 2 drugs using Cell Signalling lysis buffer. Phosphoprotein expression was explored using PathScan arrays (duplicate spots, n=3). Spot intensity was quantified through ImageJ software and data were expressed as relative mean ± SEM.

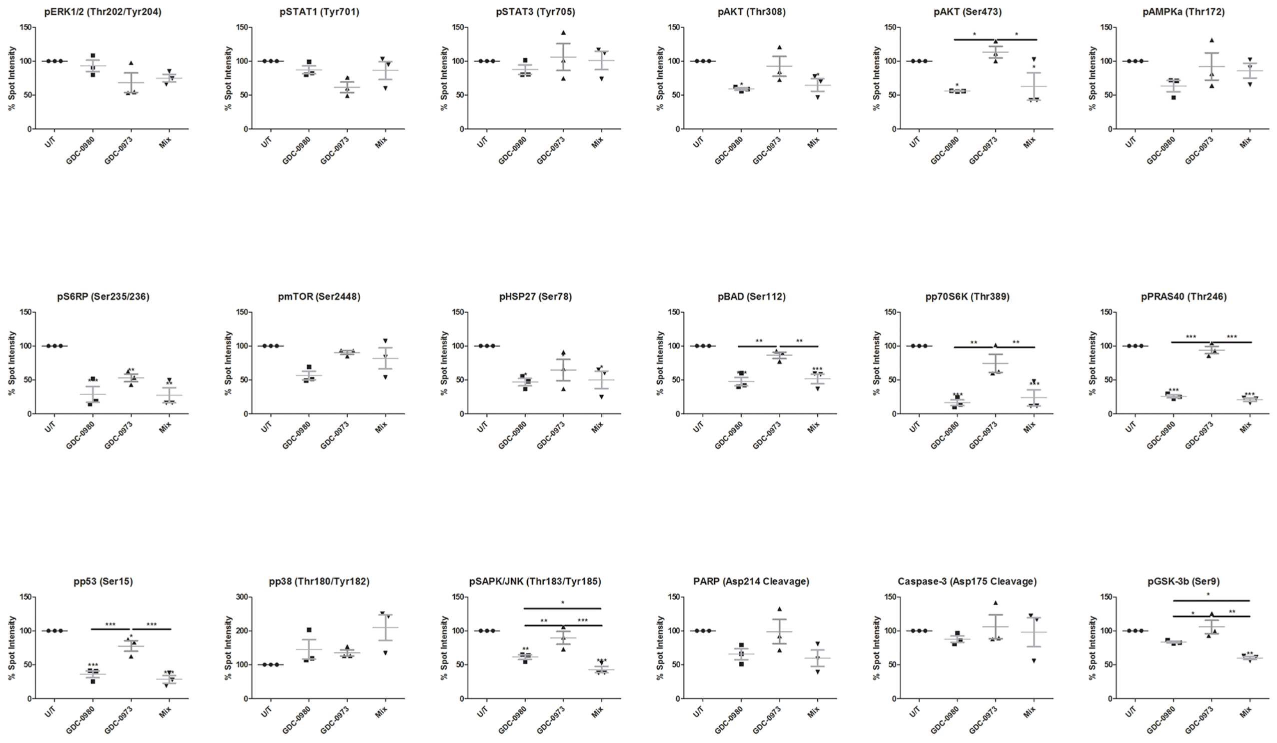

Supplementary Figure S8: Effect of GDC-0980 and/or GDC-0973 treatment on phosphoprotein expression in A549 cells. Protein was isolated from A549 cells untreated (4hrs) and treated with GDC-0980 (1µM, 4hrs), GDC-0973 (1µM, 4hrs) and a 1:1 mix of the 2 drugs using Cell Signalling lysis buffer. Phosphoprotein expression was explored using PathScan arrays (duplicate spots, n=3). Spot intensity was quantified through ImageJ software and data were expressed as relative mean ± SEM.

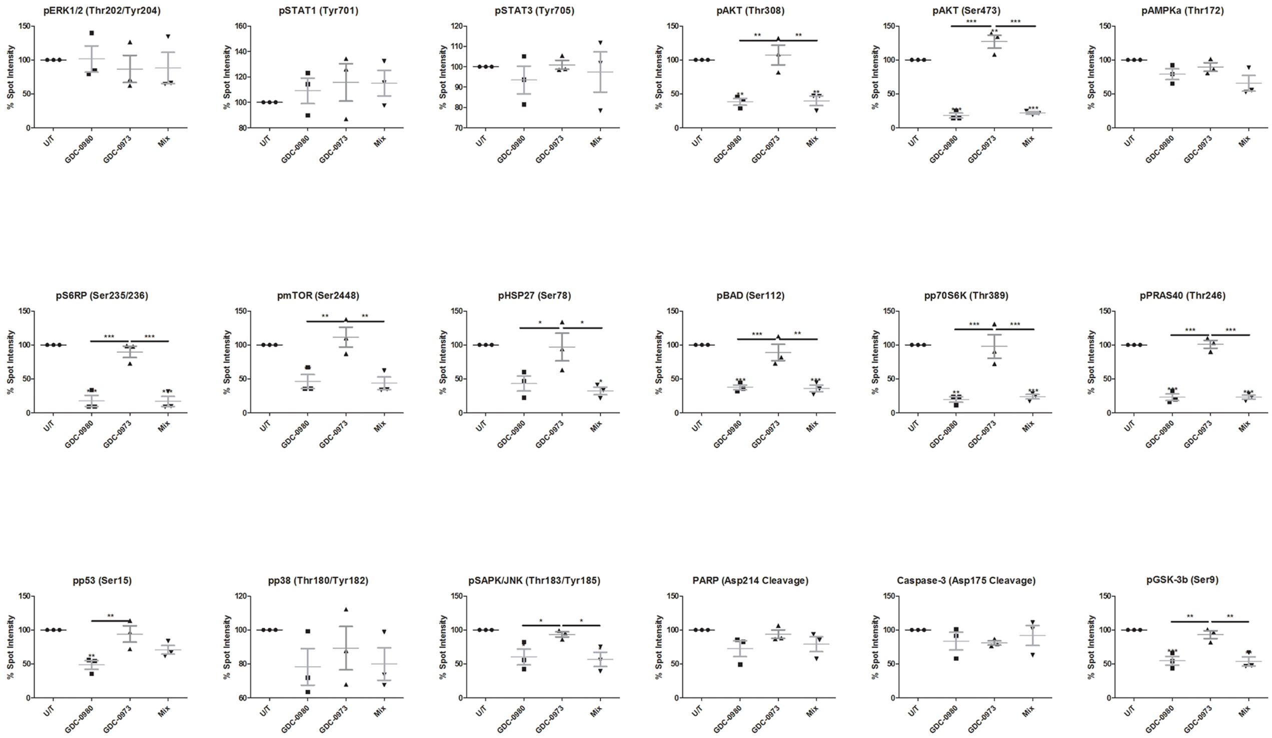

Supplementary Figure S9: Effect of GDC-0980 and/or GDC-0973 treatment on phosphoprotein expression in H1975 cells. Protein was isolated from H1975 cells untreated (4hrs) and treated with GDC-0980 (1µM, 4hrs), GDC-0973 (1µM, 4hrs) and a 1:1 mix of the 2 drugs using Cell Signalling lysis buffer. Phosphoprotein expression was explored using PathScan arrays (duplicate spots, n=3). Spot intensity was quantified through ImageJ software and data were expressed as relative mean ± SEM.

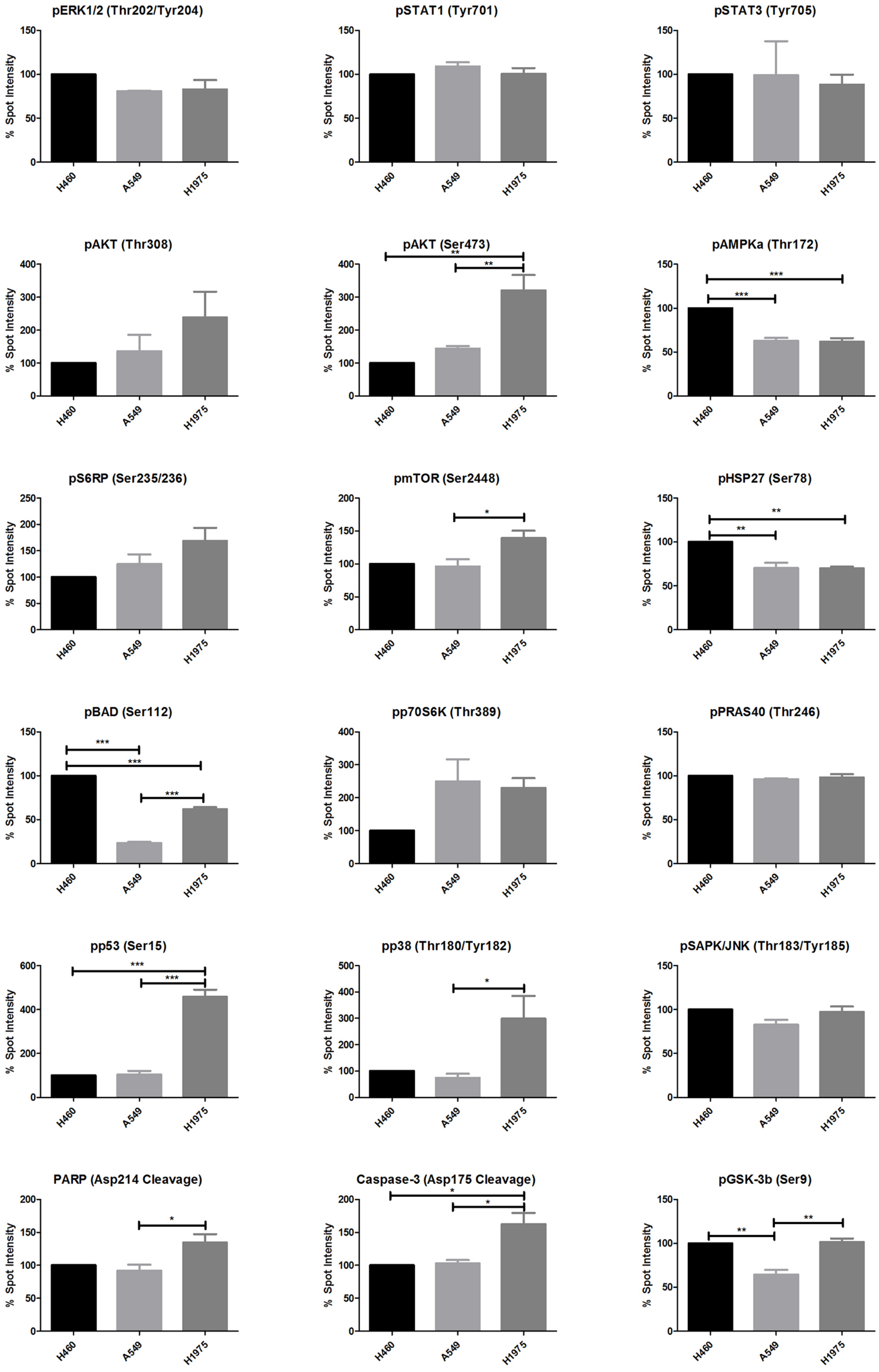

Supplementary Figure S10: Baseline phosphoprotein expression in H460, A549 and H1975 cells. Protein was isolated from H460, A549 and H1975 cells (untreated) using Cell Signalling lysis buffer. Phosphoprotein expression was explored using PathScan arrays (duplicate spots, n=3). Spot intensity was quantified through ImageJ software and data were expressed as relative mean ± SEM. */**/***: $p<0.05/0.01/0.001$ respectively, from one way ANOVA with Tukey test.

Supplementary Table S1: Fluidigm gene panel 13-gene. 175 Irish NSCLC tumour samples were profiled by Fluidigm Technology, using an allele-specific test covering 13 genes (150 mutations).

See Supplementary File 1
