# Supplementary material for: In pursuit of synergy: An investigation of the PI3K/mTOR/MEK co-targeted inhibition strategy in NSCLC
Source: Oncotarget. 2016 Oct 19;7(48):79526–43. doi: 10.18632/oncotarget.12755 (PMC5346733; doi:10.18632/oncotarget.12755)
Supplement: Supplementary file 2 [file oncotarget-07-79526-s002.docx]

**Supp. Table 1: Fluidigm gene panel 13-gene:**

| Gene | COSMIC ID |  | cDNA mutation |  | AA mutation |
| --- | --- | --- | --- | --- | --- |
| EGFR | 6252 |  | 2155 G>A |  | G719S |
| EGFR | 6253 |  | 2155 G>T |  | G719C |
| EGFR | 6239 |  | 2156 G>C |  | G719A |
| EGFR | 26038 |  | 2233_2247del15 |  | K745_E749del |
| EGFR | 13550 |  | 2235_2248>AATTC |  | E746_A750>IP |
| EGFR | 6223 |  | 2235_2249del15 |  | E746_A750del |
| EGFR | 13552 |  | 2235_2251>AATTC |  | E746_T751>IP |
| EGFR | 13551 |  | 2235_2252>AAT |  | E746_T751>I |
| EGFR | 12385 |  | 2235_2255>AAT |  | E746_S752>I |
| EGFR | 12413 |  | 2236_2248>AGAC |  | E746_A750>RP |
| EGFR | 6225 |  | 2236_2250del15 |  | E746_A750del |
| EGFR | 12728 |  | 2236_2253del18 |  | E746_T751del |
| EGFR | 12678 |  | 2237_2251del15 |  | E746_T751>A |
| EGFR | 12386 |  | 2237_2252>T |  | E746_T751>V |
| EGFR | 12416 |  | 2237_2253>TTGCT |  | E746_T751>VA |
| EGFR | 12367 |  | 2237_2254del18 |  | E746_S752>A |
| EGFR | 12384 |  | 2237_2255>T |  | E746_S752>V |
| EGFR | 18427 |  | 2237_2257>TCT |  | E746_P753>VS |
| EGFR | 12422 |  | 2238_2248>GC |  | L747_A750>P |
| EGFR | 23571 |  | 2238_2252del15 |  | L747_T751del |
| EGFR | 12419 |  | 2238_2252>GCA |  | L747_T751>Q |
| EGFR | 6220 |  | 2238_2255del18 |  | E746_S752>D |
| EGFR | 6218 |  | 2239_2247del9 |  | L747_E749del |
| EGFR | 12382 |  | 2239_2248TTAAGAGAAG>C |  | L747_A750>P |
| EGFR | 12383 |  | 2239_2251>C |  | L747_T751>P |
| EGFR | 6254 |  | 2239_2253del15 |  | L747_T751del |
| EGFR | 6255 |  | 2239_2256del18 |  | L747_S752del |
| EGFR | 12403 |  | 2239_2256>CAA |  | L747_S752>Q |
| EGFR | 12387 |  | 2239_2258>CA |  | L747_P753>Q |
| EGFR | 6210 |  | 2240_2251del12 |  | L747_T751>S |
| EGFR | 12369 |  | 2240_2254del15 |  | L747_T751del |
| EGFR | 12370 |  | 2240_2257del18 |  | L747_P753>S |
| EGFR | 13556 |  | 2253_2276del24 |  | S752_I759del |
| EGFR | 6241 |  | 2303 G>T |  | S768I |
| EGFR | 12376 |  | 2307_2308 ins 9(gccagcgtg) |  | V769_D770insASV |
| EGFR | 13558 |  | 2309_2310complex(ac>ccagcgtggat) |  | V769_D770insASV |
| EGFR | 12378 |  | 2310_2311 ins GGT |  | D770_N771insG |
| EGFR | 13428 |  | 2311_2312 ins 9(gcgtggaca) |  | D770_N771insSVD |
| EGFR | 12377 |  | 2319_2320 ins CAC |  | H773_V774insH |
| EGFR | 6240 |  | 2369 C>T |  | T790M |
| EGFR | 6224 |  | 2573 T>G |  | L858R |
| EGFR | 12429 |  | 2573-2574TG>GT |  | L858R |

| Gene | COSMIC ID | cDNA mutation | AA mutation |
| --- | --- | --- | --- |
| EGFR | 6213 | 2582 T>A | L861Q |
| PIK3CA | 746 | c.263G>A | R88Q |
| PIK3CA | 754 | c.1035T>A | N345K |
| PIK3CA | 757 | c.1258T>C | C420R |
| PIK3CA | 760 | c.1624G>A | E542K |
| PIK3CA | 763 | c.1633G>A | E545K |
| PIK3CA | 12458 | c.1634A>C | E545A |
| PIK3CA | 764 | c.1634A>G | E545G |
| PIK3CA | 765 | c.1635G>T | E545D |
| PIK3CA | 766 | c.1636C>A | Q546K |
| PIK3CA | 6147 | c.1636C>G | Q546E |
| PIK3CA | 12459 | c.1637A>G | Q546R |
| PIK3CA | 25041 | c.1637A>T | Q546L |
| PIK3CA | 773 | c.3129G>T | M1043I |
| PIK3CA | 12591 | c.3127A>G | M1043V |
| PIK3CA | 776 | c.3140A>T | H1047L |
| PIK3CA | 775 | c.3140A>G | H1047R |
| PIK3CA | 774 | c.3139C>T | H1047Y |
| PIK3CA | 12597 | c.3145G>C | G1049R |
| KRAS | 522 | c.35G>C | G12A |
| KRAS | 516 | c.34G>T | G12C |
| KRAS | 521 | c.35G>A | G12D |
| KRAS | 517 | c.34G>A | G12S |
| KRAS | 518 | c.34G>C | G12R |
| KRAS | 520 | c.35G>T | G12V |
| KRAS | 532 | c.38G>A | G13D |
| KRAS | 512 | c.34_35GG>TT | G12F |
| KRAS | 533 | c.38G>C | G13A |
| KRAS | 527 | c.37G>T | G13C |
| KRAS | 529 | c.37G>C | G13R |
| KRAS | 528 | c.37G>A | G13S |
| KRAS | 534 | c.38G>T | G13V |
| KRAS | 554 | c.183A>C | Q61H |
| KRAS | 555 | c.183A>T | Q61H |
| KRAS | 549 | c.181C>A | Q61K |
| KRAS | 553 | c.182A>T | Q61L |
| KRAS | 552 | c.182A>G | Q61R |
| BRAF | 473 | c.1798_1799GT>AA | V600K |
| BRAF | 476 | c.1799T>A | V600E |
| NRAS | 565 | c.35G>C | G12A |
| NRAS | 562 | c.34G>T | G12C |
| NRAS | 561 | c.34G>C | G12R |
| NRAS | 563 | c.34G>A | G12S |
| NRAS | 566 | c.35G>T | G12V |
| NRAS | 564 | c.35G>A | G12D |
| NRAS | 575 | c.38G>C | G13A |
| NRAS | 570 | c.37G>T | G13C |

| Gene | COSMIC ID | cDNA mutation | AA mutation |
| --- | --- | --- | --- |
| NRAS | 573 | c.38G>A | G13D |
| NRAS | 569 | c.37G>C | G13R |
| NRAS | 574 | c.38G>T | G13V |
| NRAS | 580 | c.181C>A | Q61K |
| NRAS | 584 | c.182A>G | Q61R |
| NRAS | 583 | c.182A>T | Q61L |
| NRAS | 582 | c.182A>C | Q61P |
| NRAS | 586 | c.183A>C | Q61H |
| NRAS | 585 | c.183A>T | Q61H |
| AKT1 | 33765 | c.49G>A | E17K |
| FLT3 | 785 | c.2503G>C | D835H |
| FLT3 | 783 | c.2503G>T | D835Y |
| FLT3 | 784 | c.2504A>T | D835V |
| FLT3 | 788 | c.2505T>G | D835E |
| HRAS | 480 | c.34G>A | G12S |
| HRAS | 481 | c.34G>T | G12C |
| HRAS | 483 | c.35G>T | G12V |
| HRAS | 484 | c.35G>A | G12D |
| HRAS | 487 | c.37G>A | G13S |
| HRAS | 486 | c.37G>C | G13R |
| HRAS | 496 | c.181C>A | Q61K |
| HRAS | 499 | c.182A>G | Q61R |
| HRAS | 498 | c.182A>T | Q61L |
| HRAS | 503 | c.183G>C | Q61Hc |
| HRAS | 502 | c.183G>T | Q61Ht |
| KIT | 1216 | c.1669T>A | W557R |
| KIT | 1219 | c.1669T>C | W557G |
| KIT | 1290 | c.1727T>C | L576P |
| KIT | 1304 | c.1924A>G | K642E |
| KIT | 12706 | c.1961T>C | V654A |
| KIT | 1311 | c.2446G>C | D816H |
| KIT | 1310 | c.2446G>T | D816Y |
| KIT | 1314 | c.2447A>T | D816V |
| MET | 710 | c.1124A>G | N375S |
| MET | 707 | c.3029C>T | T1010I |
| MET | 699 | c.3743A>G | Y1248C |
| MET | 700 | c.3757T>G | Y1253D |
| JAK2 | 12600 | c.1849G>T | V617F |
| MYD88 | 85940 | c.794T>C | L256P |
| ERBB2 | 14060 | c.2264T>C | L755S |
| ERBB2 | 683 | c.2263_2264TT>CC | L755P |
| ERBB2 | 14062 | c.2329G>T | L777L |
